# Temporal bone marrow of the rat and its connections to the inner ear

**DOI:** 10.3389/fneur.2024.1386654

**Published:** 2024-05-16

**Authors:** Paola Perin, Daniele Cossellu, Elisa Vivado, Laura Batti, Ivana Gantar, Fabian F. Voigt, Roberto Pizzala

**Affiliations:** ^1^Department of Brain and Behaviour Sciences, University of Pavia, Pavia, Italy; ^2^Department of Molecular Medicine, University of Pavia, Pavia, Italy; ^3^Wyss Center for Bio and Neuro Engineering, Geneva, Switzerland; ^4^Department of Molecular and Cellular Biology, Harvard University, Cambridge, MA, United States

**Keywords:** temporal bone, inner ear, bone marrow, tissue clearing, lightsheet microscopy, microCT

## Abstract

Calvarial bone marrow has been found to be central in the brain immune response, being connected to the dura through channels which allow leukocyte trafficking. Temporal bone marrow is thought to play important roles in relation to the inner ear, but is still largely uncharacterized, given this bone complex anatomy. We characterized the geometry and connectivity of rat temporal bone marrow using lightsheet imaging of cleared samples and microCT. Bone marrow was identified in cleared tissue by cellular content (and in particular by the presence of megakaryocytes); since air-filled cavities are absent in rodents, marrow clusters could be recognized in microCT scans by their geometry. In cleared petrosal bone, autofluorescence allowed delineation of the otic capsule layers. Within the endochondral layer, bone marrow was observed in association to the cochlear base and vestibule, and to the cochlear apex. Cochlear apex endochondral marrow (CAEM) was a separated cluster from the remaining endochondral marrow, which was therefore defined as “vestibular endochondral marrow” (VEM). A much larger marrow island (petrosal non-endochondral marrow, PNEM) extended outside the otic capsule surrounding semicircular canal arms. PNEM was mainly connected to the dura, through bone channels similar to those of calvarial bone, and only a few channels were directed toward the canal periosteum. On the contrary, endochondral bone marrow was well connected to the labyrinth through vascular loops (directed to the spiral ligament for CAEM and to the bony labyrinth periosteum for VEM), and to dural sinuses. In addition, CAEM was also connected to the tensor tympani fossa of the middle ear and VEM to the endolymphatic sac. Endochondral marrow was made up of small lobules connected to each other and to other structures by channels lined by elongated macrophages, whereas PNEM displayed larger lobules connected by channels with a sparse macrophage population. Our data suggest that the rat inner ear is surrounded by bone marrow at the junctions with middle ear and brain, most likely with “customs” role, restricting pathogen spread; a second marrow network with different structural features is found within the endochondral bone layer of the otic capsule and may play different functional roles.

## Introduction

Once believed to be immunologically isolated, the inner ear has been found to host a full cohort of resident and migrant immune cells [reviewed in (1)] like the brain. The inner ear and brain display several similar immune problems and solutions (e.g., swelling-induced compression, unique antigens, nonreplaceable information-critical cells, vascular barriers, local fluid compartments). In brain immunology, cranial bone marrow has recently been acknowledged as a major player, given its connections to the dura through bone channels allowing bidirectional transfer of immune cells and molecules [reviewed in (2)]. Temporal bone marrow is also known to play a major local immune role in the middle ear (where it produces immune cells related to otitis responses, which can therefore occur without appreciable systemic reactions) (3) and around the endolymphatic sac (where it changes morphologically (4) and functionally (5) in response to immune challenges to the ear). In both cases, temporal bone marrow was found to directly connect to its target (middle ear mucosa and vestibular aqueduct, respectively) through bone channels. In humans, temporal bone marrow is most commonly found in the mastoid and petrosal apex (6), where it can show pathological involvement (6–9). However, its interindividual variability, including the commixtion of marrow-filled, air-filled, and fibrotic cells, has so far limited studies of its functional connections. In rodent models, the absence of pneumatization (10) makes the analysis and interpretation of marrow distribution and connectivity patterns clearer; however, to our knowledge, a full characterization of temporal bone marrow is also lacking for rodents.

Temporal bone 3D microanatomy is undergoing a renewed interest thanks to the availability of novel X-ray-based and fluorescence-based nondestructive methods of imaging (11). Although both imaging approaches produce similar results, fluorescence-based methods based on tissue clearing (12) allow labeling of cell populations or vascular and matrix components, which is essential for understanding physiological and pathological processes as well as immune cell distribution and interactions (13–15). In this study we mapped the 3D distribution of rat temporal bone marrow and its bone and vascular connections to the inner ear, dural and middle ear spaces, by employing iDISCO+ (16) based tissue clearing with immunostaining (13) and microCT.

## Materials and methods

### Animals

Experiments were performed on 16 healthy inbred Wistar rats of either sex, for a total of 25 temporal bones (Table 1). Animals were housed with 12 h/12 h light/dark cycle, food and water provided *ad libitum*; sacrifices were performed during the light phase. This study was carried out in accordance with the recommendations of Act 26/2014, Italian Ministry of Health. The protocol (number 155/2017-PR) was approved by the Italian Ministry of Health and University of Pavia Animal Welfare Office (OPBA). All efforts were made to minimize the number of animals used and animal suffering. Three samples (R3, R8b and R16a) were used for detailed 3D morphological reconstruction of the bony and membrane labyrinth and the relative segmentations were published as datasets (17). Throughout the paper, terminology of temporal bone structures follows that by (18).

**Table 1:** List of samples with age, sex, and labeling.

Rat ID	Age (days)	Sex	Brain present	CAEM	PNEM	VEM	Labeling
R1a	111	F	Yes	−	−	+	Autofluorescence + Iba1 (647)
R1b	111	F	Yes	+	−	+	Autofluorescence + Iba1 (647)
R2	95	F	Yes	+	−	+	Autofluorescence (488) + Iba1 (647)
R3	92	F	No	+	+	+	SMA (565n) + Autofluorescence (647)
R4a	98	F	Yes	+	−	−	Autofluorescence (488) + Iba1 (555) + TO-PRO (647)
R4b	98	F	Yes	+	−	−	Autofluorescence (488) + Iba1 (555) + TO-PRO (647)
R5a	98	M	Yes	+	−	−	Autofluorescence (488) + Iba1 (555) + TO-PRO (647)
R5b	98	M	Yes	+	−	−	Autofluorescence (488) + Iba1 (555) + TO-PRO (647)
R6	98	F	Yes	+	−	+	Autofluorescence (488) + SMA (565n)
R7a	98	F	Yes	+	+	+	Autofluorescence (488) + SMA (565n) + Col-IV (647n)
R7b	98	F	Yes	+	+	−	Autofluorescence (488) + SMA (565n) + Col-IV (647n)
R8a	494	M	Yes	+	−	+	Autofluorescence (488) + SMA (565n) + Autofluorescence (647)
R8b	494	M	Yes	+	−	+	Autofluorescence (488) + SMA (565n) + Autofluorescence (647)
R9a	64	M	Yes	+	+	+	Autofluorescence (488) + vWF (555) + Col-IV (647n)
R9b	64	M	Yes	+	+	+	Autofluorescence (488) + vWF (555) + Col-IV (647n)
R10a	79	F	Yes	+	+	−	Autofluorescence (488) + TO-PRO (647)
R11a	79	M	Yes	+	+	+	SMA (488) + vWF (555) + TO-PRO (647)
R11b	79	M	Yes	+	+	−	SMA (488) + vWF (555) + TO-PRO (647)
R12	741	F	No	none	−	+	SMA (488) + Col-IV (555) + vWF (555) + TO-PRO (647)
R13	466	F	No	none	−	−	Autofluorescence (488)
R14	129	F	No	−	−	−	ColIV (488)
R15a	79	M	No	+	+	+	ColIV (488) + vWF(555)
R15b	79	M	No	+	−	+	Autofluorescence (488) + ColIV(555)
R16a	79	F	Yes	+	+	+	Lugol microCT
R16b	79	F	Yes	+	+	+	Lugol microCT

Temporal bones from the same animal are indicated as **a** (left) and **b** (right); nanobodies (see Methods) are indicated with **n** the letters a, b, and n should be bold. Some samples (marked with -) did not allow a complete reconstruction of bone marrow clusters because of bone trimming, lightsheet artifacts or insufficient tissue clearing. In a few samples (marked as “none”), it was impossible to find the corresponding cluster.

### Surgery and sample preparation

Rats were anesthetized as in (13), and transcardially perfused with cold heparinated PBS through a peristaltic pump. After complete blood removal (assessed by mucosal whitening) rats were perfused with 4% PFA until optimal tissue hardening and decapitated. Skin and muscle were removed from the head, which was post-fixed for 24 h and subsequently trimmed removing most parietal and occipital bone. Fixed trimmed heads were cryoprotected in 30% sucrose for one week. After cryoprotection, heads were decalcified with buffered 5% EDTA (with shaking, daily changes) until softening of the squamous temporal bone (3–4 weeks at room temperature or 2–3 weeks at 37°C). For rat R8, microwave-aided decalcification with buffered 5% EDTA was performed (8 h for 3 days at 40°C, with change every hour, using Milestone MLS 1200 Mega high-performance microwave). In some cases, the brain was used for different experiments, and the temporal bone was dissected free, whereas in others the whole posterior part of the head (cranium and brain, sectioned at the midbrain level) was processed, allowing access to the dura and its sinuses in their entirety.

### Tissue clearing

Tissue clearing was performed as in (19); samples were immunolabeled with rabbit anti- Collagen IV (ColIV; Abcam ab6586, 1:200) sheep anti-von Willebrand factor (vWf; Genetex GTX74137, 1:100), mouse anti-smooth muscle actin (SMA; Abcam amab7817, 1:200), and rabbit anti-Iba1 (WAKO 019–19,741, 1:200); secondary antibodies (Invitrogen Donkey anti-sheep, anti-rabbit and anti-mouse conjugated with Alexa 488, 555 or 647) or nanobodies (20) (Synaptic System nanobodies anti-mouse and anti-rabbit conjugated with ATTO Fluor 565 and 647 dyes) were all used at 1:200. Incubations with nanobodies required half the time than with regular antibodies. TO-PRO labeling (2 h, Sigma T3605, 1:500 or 1:1000) was used to stain cell nuclei at the end of secondary antibody incubation. Unfortunately, several microvascular markers did not label our cleared samples (PECAM-1: Santa Cruz mouse anti-mouse and predicted rat, sc-376764; RECA-1: abcam, mouse anti-rat, ab9774 and ab264524, PROX-1: Sigma, mouse anti-rat, p0089; LYVE-1: R&D systems, sheep anti-rat, af7939); therefore, lymphatic vessels and marrow sinusoids were not traced.

### Microscopy

Cleared samples were imaged using a custom-made mesoscale selective plane-illumination microscope [mesoSPIM (21)] system at the Brain Research Institute, University of Zurich and at the Wyss Center for Bio and Neuroengineering in Geneva, Switzerland, which allowed imaging of sample in its entirety (travel range 44 × 44 × 100 mm), providing near-isotropic resolution 3D datasets. Higher resolution scans were made on a CLARITY-optimized light-sheet microscope [COLM (22)] at the Wyss Center for Bio and Neuroengineering in Geneva, Switzerland, as in (19).

#### mesoSPIM

Scans performed at UZH had the same protocols and equipment described in (19). For scans performed at Wyss, after staining and clearing, samples were attached to a custom 3D-printed holder, then submerged in a size-matched quartz cuvette (Portmann Instruments) filled with dibenzyl ether (DBE, nd = 1.562) and imaged using a home-built mesoSPIM. The microscope consists of a dual-sided excitation path using a fiber coupled multiline laser combiner (405, 488, 561, and 647 nm, Toptica MLE) and a detection path comprising a 42 Olympus MVX-10 zoom microscope with a 1× objective (Olympus MVPLAPO 1×), a filter wheel (Ludl 96A350), and a scientific CMOS (sCMOS) camera (Hamamatsu Orca Flash 4.0 V3). The excitation paths also contain galvo scanners for light sheet generation and reduction of shadow artifacts due to absorption of the light sheet. In addition, the beam waist is scanned using electrically tunable lenses (ETL, Optotune EL-16-40-5D-TC-L) synchronized with the rolling shutter of the sCMOS camera. This axially scanned light-sheet mode (ASLM) leads to a uniform axial resolution across the field-of-view (FOV) of 5 μm. Image acquisition is done using custom software written in Python. Excitation wavelengths were set at 488, 561, and 647 nm, with an emission 530/40 nm bandpass filter, 593/40 bandpass and 663 LP filter, respectively, (BrightLine HC, AHF). All acquisitions were made from only one angle in a multitile setup (2 × 2) using the closest light sheet. Z-stacks (2048 × 2048 pixels image stacks) were acquired with zoom set at from 0.8X to 4X resulting in xy pixel sizes from 8.2 to 1.6 μm, at a quasi-isotropic Z spacing (1, 3, 4, 5, or 8 μm).

#### COLM

Light-sheet imaging was performed using a customized version of the Clarity Optimized Light-sheet Microscope (COLM) at the Wyss Center Advanced Light-sheet Imaging Center, Geneva. The samples were illuminated by one of the two digitally scanned light sheets, using a 488 nm, 561 nm and 647 nm wavelength laser. Emitted fluorescence was collected by 4X XLFLUOR4X N.A. 0.28 filtered (525/50 nm, 609/54 nm and 680/42 Semrock BrightLine HC) and imaged on an Orca-Flash 4.0 LT digital CMOS camera at 4 fps, in rolling shutter mode. Z-stacks were acquired at 5 μm spacing with a zoom set at 4x resulting in an in-plane pixel size of 1.44 μm (2048 × 2048 pixels). A self-adaptive positioning system of the light sheets across z-stacks acquisition ensured optimal image quality over the whole thickness of the region of interest.

### Micro-computed tomography (microCT)

The microCT sample was counterstained with iodine by Lugol (Merck 1.0926.1000) immersion until solution clearing, for soft tissue staining. MicroCT observations were performed on the local core facility SkyScan 1,276 CMOS (Bruker, Kontich, Belgium) using step and shoot acquisition with a 0.2-degree rotation step. Images were reconstructed using the software Bruker Nrecon 1.7.4 with ring artifact correction and beam hardening, and Gaussian smoothing to limit rogue voxel artifacts (23). The sample was observed with source settings of 85 kV/47 μA and 1 mm Al filter. Images were 3272 × 3092 × 2189 pixels, at a resolution of 6.4 μm.

### Image analysis

Before any analysis, lightsheet image stacks were processed to reduce z-axis anisotropy and lightsheet stripe artifacts. For z-axis anisotropy, image stacks were deconvolved using the MATLAB script from (24), with a NA of 0.14 for MesoSPIM (21) and 0.28 for COLM (22) and the refractive index of DBE (1.562) and scaled to isotropy with FIJI (25). For stripe artifact minimization, we filtered datasets with the SPIM-Matlab-Destripe-Deshake macro from (26) where necessary.

After deconvolution and destriping, segmentation of inner ear labyrinth, blood vessels, bone marrow clusters and bone channels was performed with FIJI (25), ITK-SNAP (27) and 3D-Slicer (28) with the following pipeline:

1. CLAHE (FIJI macro, optional);
2. Top hat filter (FIJI, only for vessels and channels);
3. Raw threshold segmentation with Otsu (FIJI macro);
4. Generation of initial segmentation mask (FIJI macro);
5. Gross manual correction of mask (FIJI or ITK-SNAP);
6. Snake segmentation optimization (ITK-SNAP or 3D-Slicer) using mask as seed;
7. Manual correction of segmentation (ITK-SNAP);
8. Iterate 6- and 7 until consensus on optimal segmentation.

This pipeline allowed segmentation of a labyrinth in a few hours; for other structures, which had a more variable shape and less sharp demarcation, time was longer. Segmentation was performed by three independent researchers and a consensus was reached upon comparisons.

In all animals, we analyzed bone marrow clusters, dural sinuses, inner ear labyrinth, bone channels, and blood vessels. Cleared tissues display autofluorescence, which is often used as a guide for identifying structures in large 3D samples (29). In our samples, autofluorescence allowed the segmentation of marrow clusters and bony and membranous labyrinths. Autofluorescence evoked by 647 nm light also allowed to delineate bone matrix differences between endochondral and endosteal bone (see Results). Given the complexity of inner ear 3D structures, in where necessary we show 3D reconstructions of the autofluorescence signal of samples R1b and R3 to clarify where the structures of interest are located.

Bone marrow was observed as granular content within bone cavities in the auto-fluorescence or TO-PRO signal. To measure volumes, we segmented bone cavities rather than the associated cellular masses, since tissue shrinking due to the iDISCO+ technique [which is estimated at about 10% (16)] could lead to marrow collapse, especially in larger lobules.

Volumes were obtained as scaled voxel counts from ITK-SNAP or 3D-Slicer; for a visualization of their spatial congruence, labyrinths were registered to each other using 3D Slicer landmark-based registration. The pipeline was as follows:

1. Point clouds were obtained from R16b bone labyrinth segmentation files, and landmarks were added with SlicerMorph pseudoLMgenerator (30);
2. The obtained landmarks were transferred to labyrinth segmentations from other samples and ALPACA (31) was applied;
3. Transform matrices were generated using SlicerIGT Fiducial Registration Wizard module (32);
4. The same transform used for labyrinth registration was applied to bone marrow.

Spatial congruence of marrow clusters in different temporal bones was observed by superimposing each cluster 3D volume reconstruction to the volume sum of all clusters (with the Union operator by 3DSlicer). Ectotympanic bone marrow volume was only quantified in 4 samples, since in most samples the bulla was opened to ease fixation and immunolabeling and was therefore incomplete. Samples with incompletely visible marrow clusters (due to excessive trimming, lightsheet artifacts or suboptimal clearing in the temporal bone region of interest) were not used for quantitative observations.

Volumes were compared between clusters and gender. Significance of differences was tested with factorial ANOVA using R (33).

Blood vessels (including dural sinuses) were visualized by ColIV labeling [which labels all basal laminae (34)], vWf [which labels vascular endothelium (35) and megakaryocytes (36)], and SMA [which labels smooth muscle (37)]. Autofluorescence at 647 nm delineated bone channels. Bone and vascular channels were traced throughout the temporal bone from mesoSPIM and COLM scans with a resolution from 1 to 4 μm; channel diameters were measured by selecting 10 random optical sections perpendicular to the channels, binarizing them, and measuring diameters of the object with circularity >0.9.

Since most structures in lightsheet stacks were visualized by the combination of bright and dark details (e.g., bright vessel wall and dark vessel lumen) and displayed convoluted pathways, in several images we employed Maxmin stack visualization, which was performed as follows (see also Supplementary Figure S1).

1. The depth region containing the structure of interest was chosen in the image stack;
2. Maximum intensity projection (MIP) was performed applying the ImageJ ZProject function over the chosen depth region (obtaining a single image showing the projection of bright structures) and false-colored red;
3. Minimum intensity projection (MinIP) was performed applying the ImageJ ZProject function over the chosen depth region (obtaining a single image showing the projection of dark structures) and false-colored cyan;
4. MIP and MinIP were combined, obtaining an image in which the dark structures were visible as dark holes on cyan background, and the bright structures were visible in red.

## Results

### Distribution of rat temporal bone marrow

**Figure 1:**
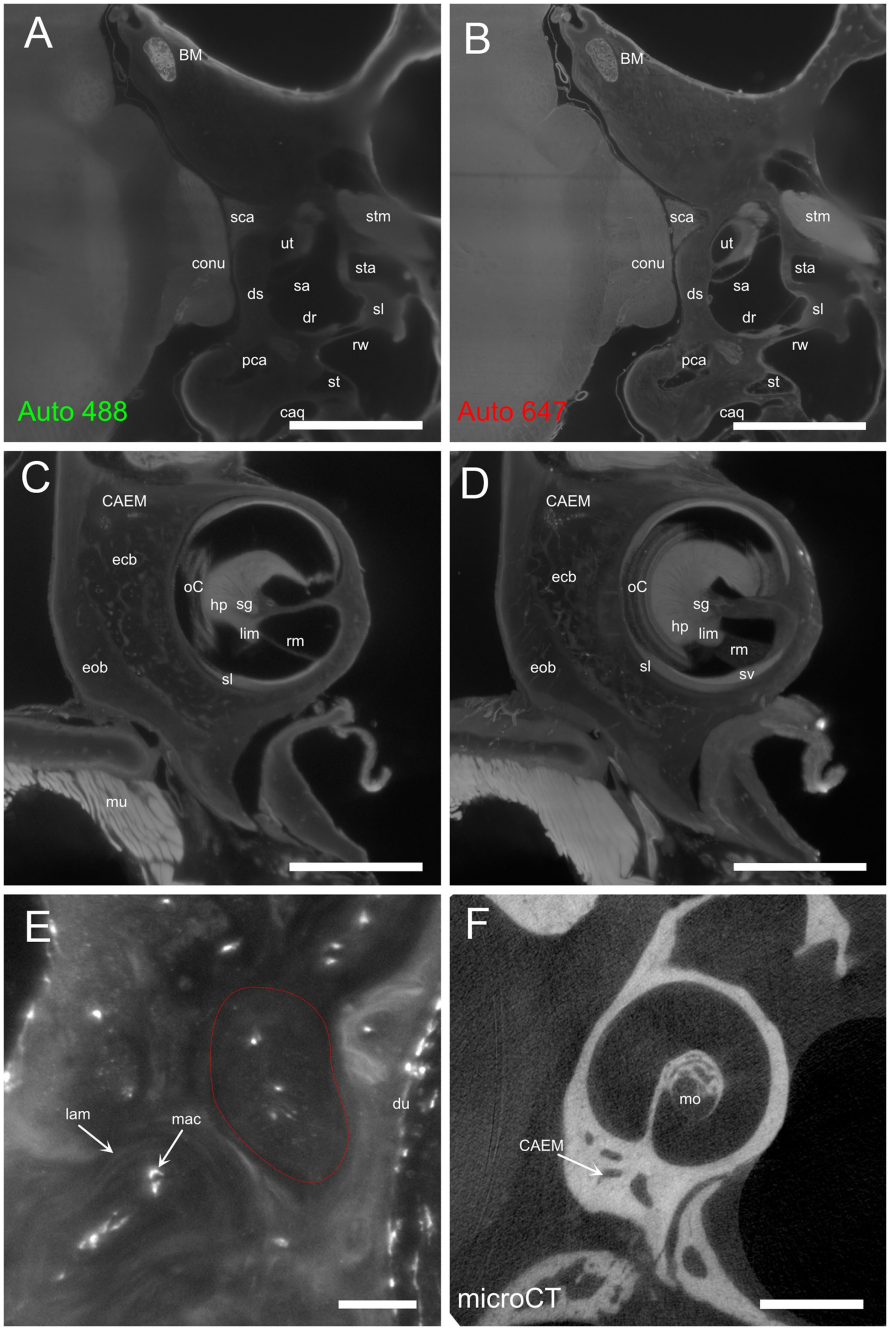
Autofluorescence signal. **(A)** Single optical section, 488 nm autofluorescence signal. **(B)** Single optical section, 647 nm autofluorescence signal, same section as **(A)**. **(C)** Single optical section showing the cochlea and surrounding bone, 647 nm autofluorescence signal. **(D)** Maximal intensity projection of a 150 μm stack around the section in **(C)**. **(E)** High-magnification detail of osteon-like structures in endochondral bone (one osteon is outlined in red). Single optical section of 647 nm signal containing Iba1+ labeling and autofluorescence signal. **(F)** Single retroprojected slice from microCT signal showing the cochlea and CAEM. Images in panels **(A–D)** are from sample R8b; image in panel **(E)** from sample R1a; image in panel **(F)** from sample R16a. Scale bars are: 100 μm for panel **(E)**, 1 mm for other panels.

Temporal bone marrow distribution could be visualized in lightsheet scans of cleared samples and microCT scans of iodine-contrasted samples (Figure 1). In cleared samples, autofluorescence allowed the segmentation of bone marrow as well as bony and membranous labyrinths. Bone marrow was well delineated using the autofluorescence signal excited at 488 nm (Figure 1A), but the autofluorescence signal excited at 647 nm also displayed local intensity variations within bone matrix (Figure 1B). Given that endochondral and membranous bone differ in collagen I and osteocalcin expression patterns (38), and that both proteins have been observed to be autofluorescent in cleared bone (14), these intensity differences appear to correlate with endochondral bone distribution. As further support of this, around the cochlea the geometry of low-autofluorescence bone (Figures 1C,D) was similar to that of endochondral bone (39) and at high resolution osteon-like layered patterns could be seen in this region (Figure 1E), consistent with the presence of osseous globuli, which predominate in the rat endochondral bone (40). On the other hand, microCT allowed to visualize the distribution of bone marrow cavities but did not allow to observe local variations of bone matrix structure or cavity contents (Figure 1F).

**Figure 2:**
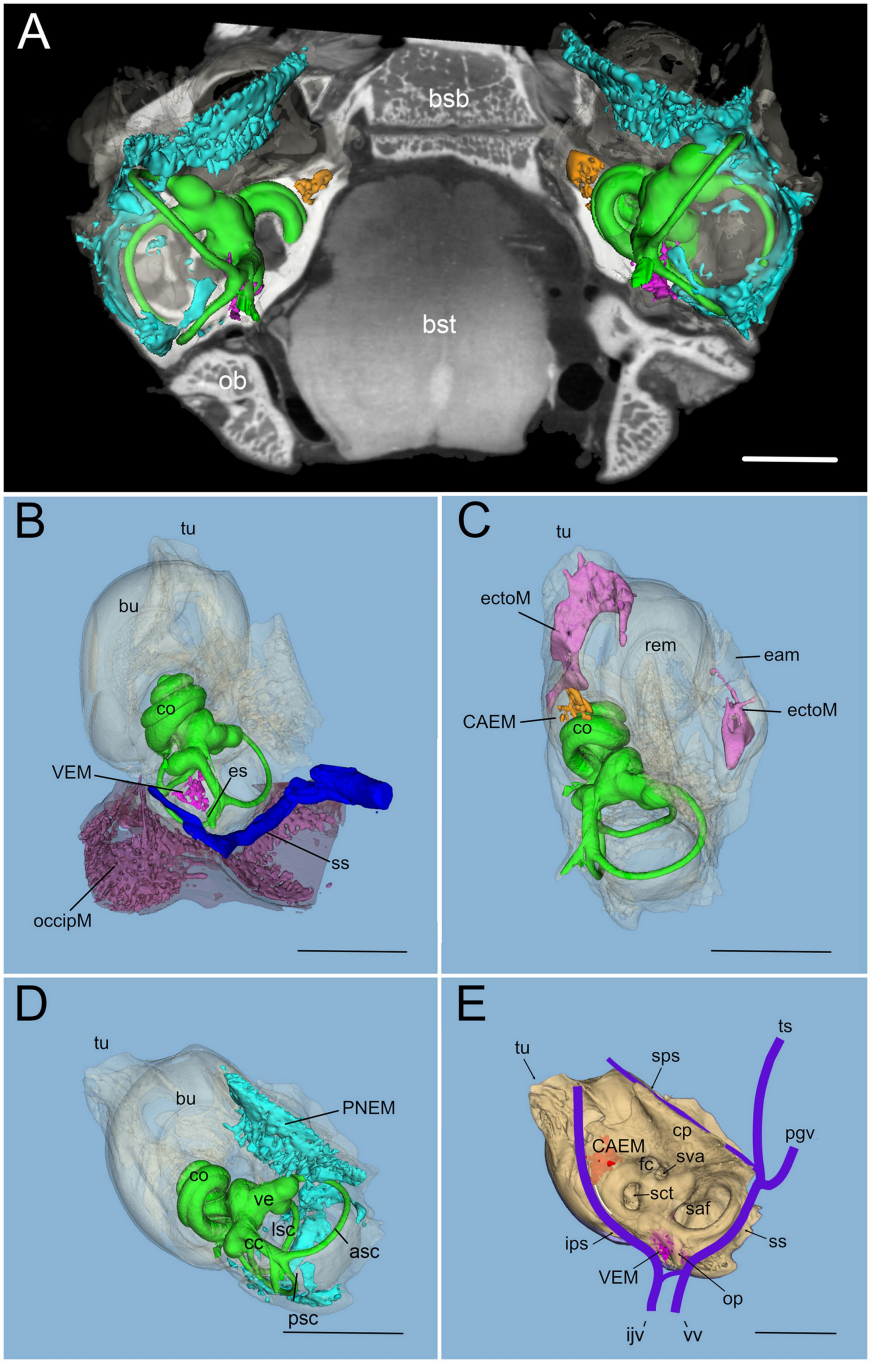
Global 3D reconstruction of rat temporal bone structures from microCT. In all panels, bone labyrinth is green, CAEM orange, VEM magenta, petrosal membranous bone marrow cyan, ectotympanic bone marrow pink, occipital bone marrow purple; temporal bone is semitransparent **(B–D)** or solid beige **(E)**. **(A)** Bilateral inner ear 3D reconstruction with bone marrow clusters superimposed on a horizontal microCT slice. Ossicles and stapedial artery are not shown. **(B)** Medial view of the temporal bone showing VEM and occipital bone marrow; occipital bone is shown in semitransparent brown, and dural sinus in blue. Anterior at the top right corner. **(C)** Dorsal view of the temporal bone showing CAEM and ectotympanic marrow (anterior at the top right corner). **(D)** Dorsomedial view of the temporal bone showing petrosal membranous bone marrow. Anterior at the top. **(E)** Dorsomedial view of the temporal bone showing the paths of dural sinuses [as in (18)]. Unions of CAEM (red) and VEM (pink) volumes from all samples are shown within bone (compare with Figure 6). White line shows the location of small lobule “trail” leaving CAEM. Anterior at the top. Panel **(A)** is a 3D reconstruction from sample R16 (a and b), other panels are reconstructions from sample R16b. All scale bars: 2 mm.

In rat, differently from human, temporal bone ossification centers do not merge into a single bone in adulthood: in particular, the rat shows a petrosal bone containing the inner ear, an ectotympanic bone forming the middle ear bulla, and a squamosal bone (18). Rat petrosal bone marrow could be located outside the otic capsule (Figures 1A,B) or within its endochondral layer (Figures 1C,D). Since petrosal marrow (Figure 2A) was contained within several separate cavities rather than in a single cavity like in calvarial bones (see Supplementary Figure S2), we called its units “clusters.”

Within petrosal endochondral bone, marrow was found clustered in a region reaching from the cochlear base to the endolymphatic sac (VEM, vestibular endochondral marrow, Figures 2B,E) and at the cochlear apex (CAEM, cochlear apex endochondral marrow, Figures 2C,E). In our data, all samples displayed separate CAEM and VEM, but in 3 cases we observed a “trail” of small marrow lobules with vascular connections to CAEM following the inferior petrosal sinus pathway around the cochlea (white line in Figure 2E). Outside endochondral bone, a large marrow cluster surrounded semicircular canal arms, extending into the crista petrosa (PNEM, petrosal non-endochondral marrow, Figure 2D). Other marrow of potential interest to inner ear responses was found in the occipital bone, across the distal portion of the endolymphatic sac (Figure 2B), and in the ectotympanic bone in association with the middle ear bulla (Figure 2C). Both ectotympanic and occipital marrows were connected to the dural sinuses which surround the petrosal bone (Figure 2E).

**Figure 3:**
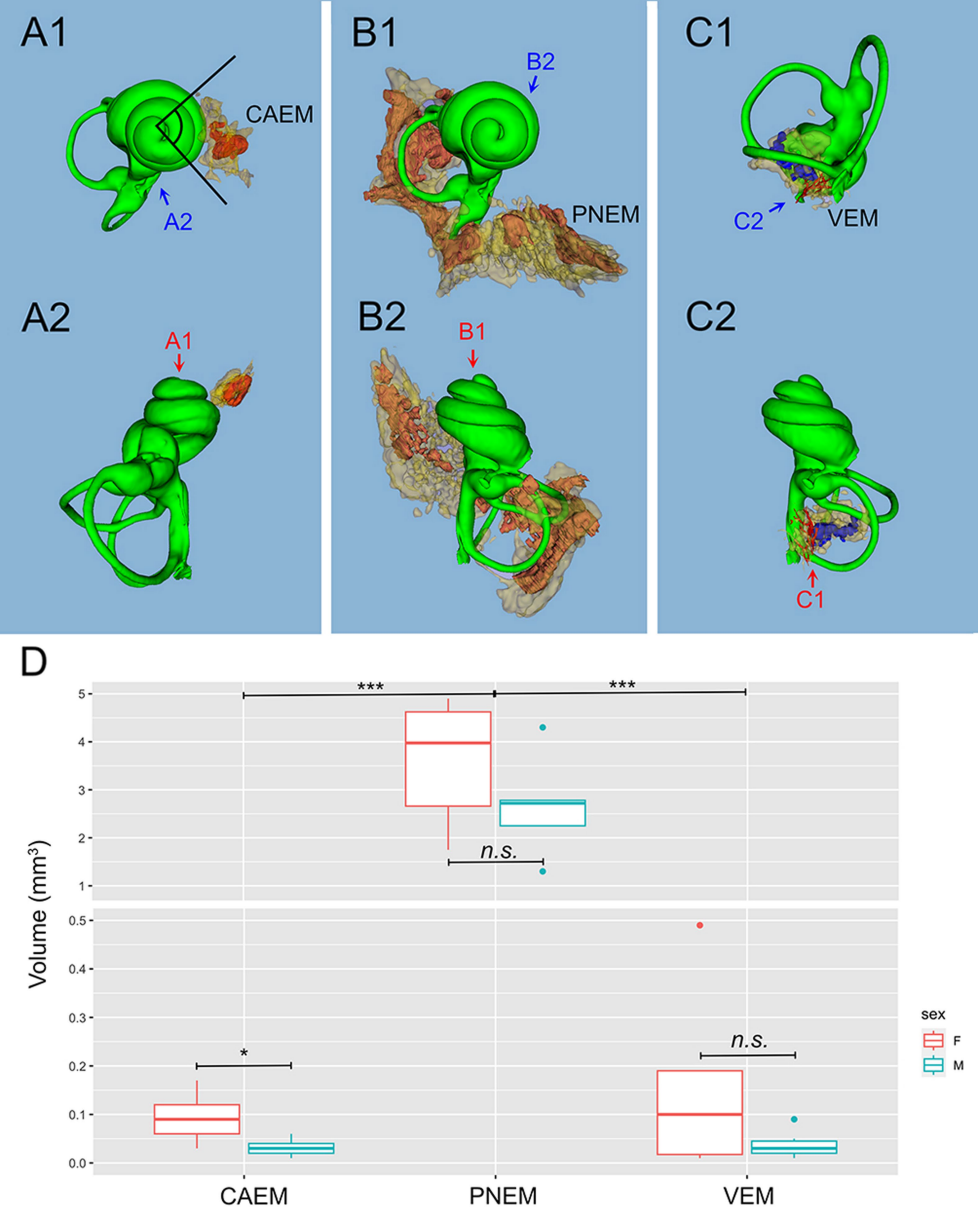
Marrow clusters position, geometry, and size. In **(A–C)**, clusters are shown in their position relative to the labyrinth, in association with the bony labyrinth of R16b (in green) for reference; the union of bone marrow volumes from all samples is shown for each cluster in semitransparent yellow; clusters from one sample (in parentheses for each cluster) are shown in solid red, except for **(C)** where reticular marrow is red and spheroidal blue. Two orthogonal views are shown for each cluster (arrows indicate relative views). **(A)** CAEM (R3). **(A1)** View from cochlear apex, **(A2)** view from ventromedial side; in 1, the angle superimposed to the cochlea is 90 degrees. **(B)** Petrosal membranous bone marrow (R3), **(B1)** view from cochlear apex, **(B2)** view from dorsomedial side. **(C)** VEM (R1b reticular, R2 spheroidal), **(C1)** view from endolymphatic sac, **(C2)** view from dorsomedial side. **(D)** Boxplot of temporal bone marrow cluster volumes. Scale is expanded below 0.5 mm3 to show sex-related differences in VEM and CAEM (ANOVA test: CAEM *p* = 0.03, PNEM *p* = 0.1, VEM *p* = 0.38).

Temporal bone marrow distribution from different animals (Figure 3) was consistent, displaying a cluster at the cochlear apex (CAEM, Figure 3A), one around semicircular canal arms, outside the otic capsule (PNEM, Figure 3B), and one around the base of the cochlea and the vestibule, extending around the crus commune (VEM, Figure 3C). Endochondral bone marrow clusters (Figure 3D), were significantly smaller than PNEM [CAEM = 0.06 ± 0.05 mm^3^ (*n* = 21), VEM = 0.09 ± 0.13 mm^3^ (*n* = 15), PNEM = 3.18 ± 1.26 mm^3^ (*n* = 11)] and displayed sex-linked volume differences (Figure 3D).

### Bone marrow features

**Figure 4:**
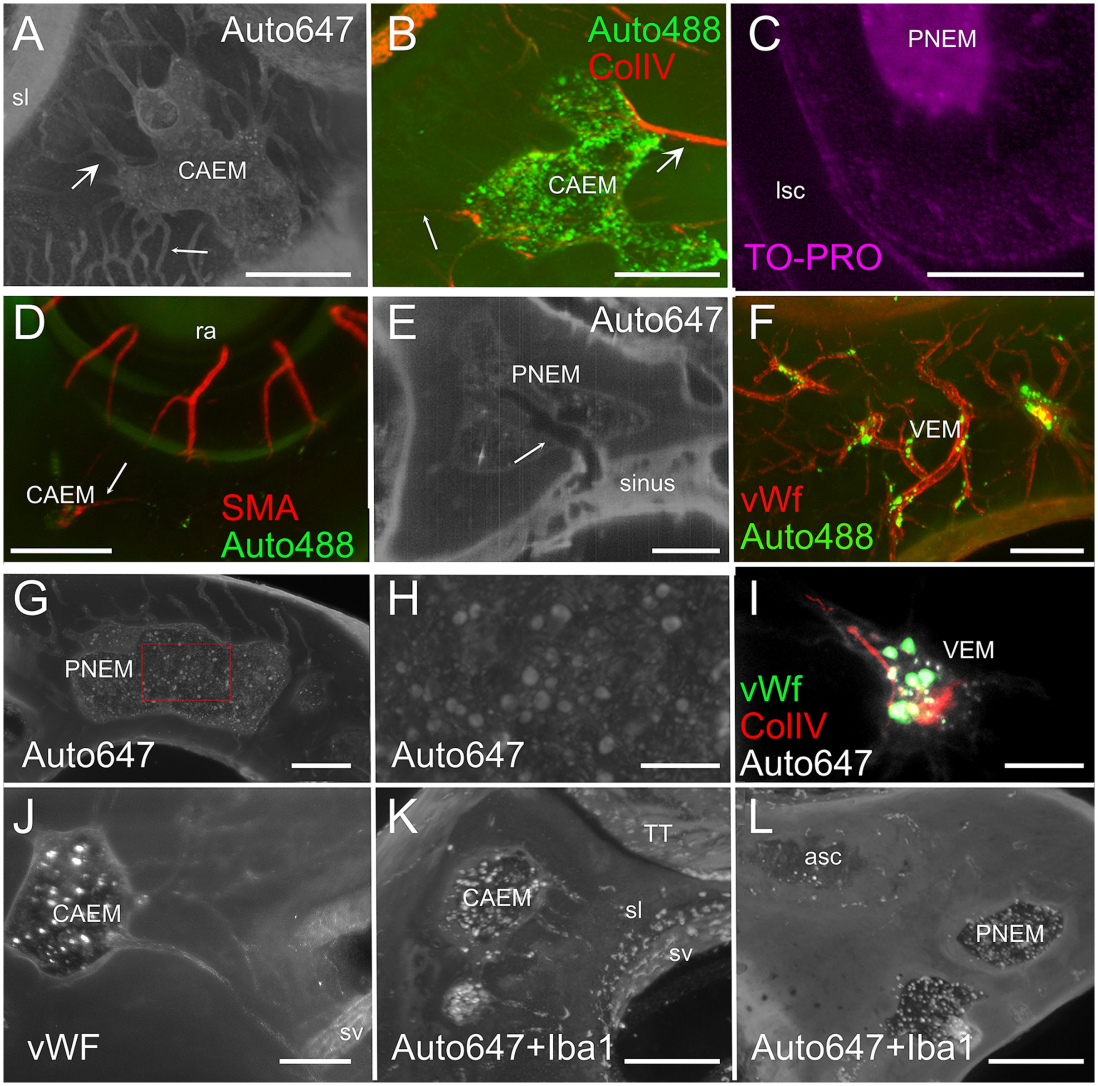
Bone marrow microstructure. **(A)** Bone channels associated to bone marrow (CAEM). Here and in panel **(B)**, small arrows indicate small channels, large arrows indicate large channels. MIP of a 120 μm stack, 647 nm autofluorescence signal. **(B)** ColIV+ blood vessels associated to bone marrow (CAEM), MIP of a 120 μm stack. Both small and large vessels are seen. Note the lower density compared to the channels in **(A)**. **(C)** TO-PRO optical slice showing cell nuclei in the bone marrow and surrounding bone. Note the absence of nuclei in the semicircular canal. **(D)** SMA+ arteriole (CAEM). MIP of a 300 μm stack. Most marrow clusters had at most one feeding arteriole (arrow). **(E)** Bone marrow diploic vein (PNEM) visible as void in marrow autofluorescence (arrow). Single optical section resliced on vein plane. **(F)** Reticular bone marrow cluster (VEM). MIP of a 300 μm stack. Green: 488 nm autofluorescence, red: vWf. **(G)** Spheroid bone marrow lobule (PNEM), 647 nm autofluorescence signal. MIP of a 100 μm stack. Channels connect marrow to the dura. **(H)** Magnification of the red rectangle in **(G)**. **(I)** vWf labeling of large cells within a reticular marrow lobule (VEM). Single optical section. **(J)** vWF cells in CAEM lobules. MIP of 125 μm stack. **(K)** Iba1+ cells in CAEM lobules. MIP of 30 μm stack. **(L)** Iba1+ cells in PNEM lobules. MIP of 30 μm stack. Lobules are connected but appear separated in this stack. Image in panel **(A)** is from R3, in **(B)** from R15b, **(C)** from R5, **(D)** from R8b, **(E,I)** from R15a, **(F,J)** from R11, **(G,H)** from R6, **(K,L)** from R1b. Scale bars are 300 μm in all panels except 100 μm in **(H,I)**.

Temporal bone marrow could be visualized (Figure 4) within the autofluorescence signal evoked at 647 nm (Figure 4A) and 488 nm (Figure 4B) as bone cavities filled with granular substance; TO-PRO labeling (Figure 4C) showed that these cavities contain a very high density of cell nuclei. The 647 nm autofluorescence signal also displayed bone channels departing from marrow clusters (Figure 4A), which included small (mean diameter 20.9 ± 4.1 μm, *n* = 635) and large (mean diameter 35.0 ± 5.3 μm, *n* = 47) populations. Some of these channels contained ColIV+ blood vessels (Figure 4B) and SMA+ arterioles (Figure 4D). Bone marrow sinusoids were not visible due to lack of selective labeling for the rat (see Methods); larger marrow diploic veins (diameter > 50 μm) were instead visible as tubular voids in marrow autofluorescence (Figure 4E).

Petrosal bone marrow displayed “spheroid” lobules (Figures 4G,H), which could be interconnected to each other or isolated, and “reticular” lobules with smaller size and irregular shape, resembling a thickening of bone vessels at their crossing (Figures 4F,I). The latter marrow lobules could only be observed in endochondral bone.

In all clusters, marrow lobules displayed very high cell density and contained cells identified as megakaryocytes (36) due to their large size (see Figures 4G,H) and labeling with vWf (Figures 4I,J), and a heterogeneously-distributed population of rounded Iba1+ cells (Figures 4K,L). The presence of Iba1+ myeloid cells and especially megakaryocytes allowed us to define these spaces within bone cavities as bone marrow.

### Cochlear apex endochondral bone marrow (CAEM)

**Figure 5:**
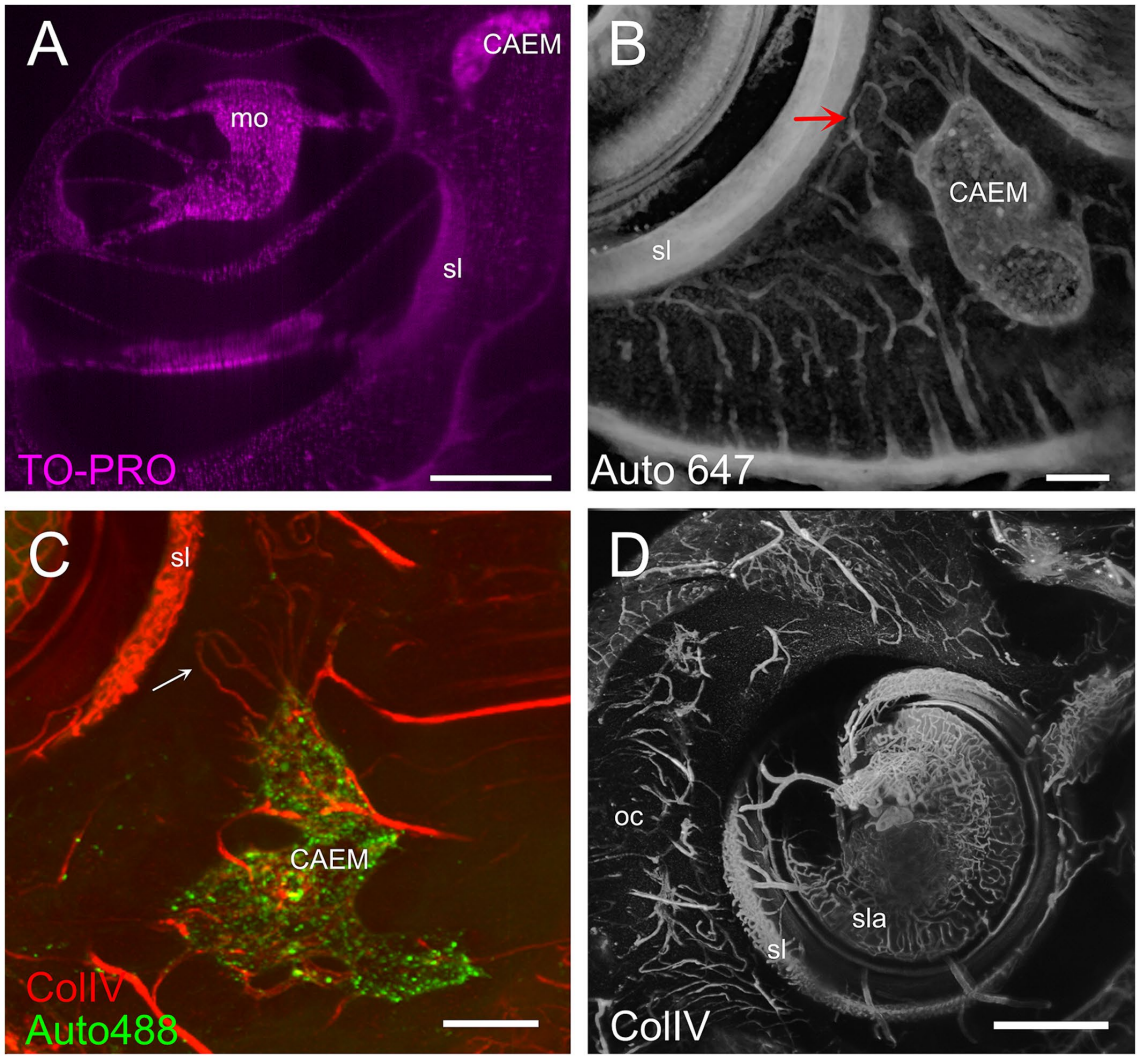
Cochlear apex endochondral marrow (CAEM). **(A)** TO-PRO optical slice showing density of cell nuclei around the cochlea. Nuclei are separable throughout the bone and cochlear structure but are too dense in CAEM to be resolved. **(B)** Bone channels, 647 nm autofluorescence signal. MIP of 80 μm stack showing a bone channel loop from CAEM coasting the spiral ligament (arrow). **(C)** Vascular loops (arrow) from bone marrow to spiral ligament. MIP from a 200 μm stack. Red: ColIV labeling; green: 488 nm autofluorescence signal. **(D)** Blood vessels in the otic capsule and cochlea. MIP from a 500 μm stack of ColIV signal. Image in panel **(A)** is from R5, **(B)** from R3, **(C,D)** from R15b. Scale bars are 500 μm in **(A,D)** and 200 μm in **(B,C)**.

**Figure 6:**
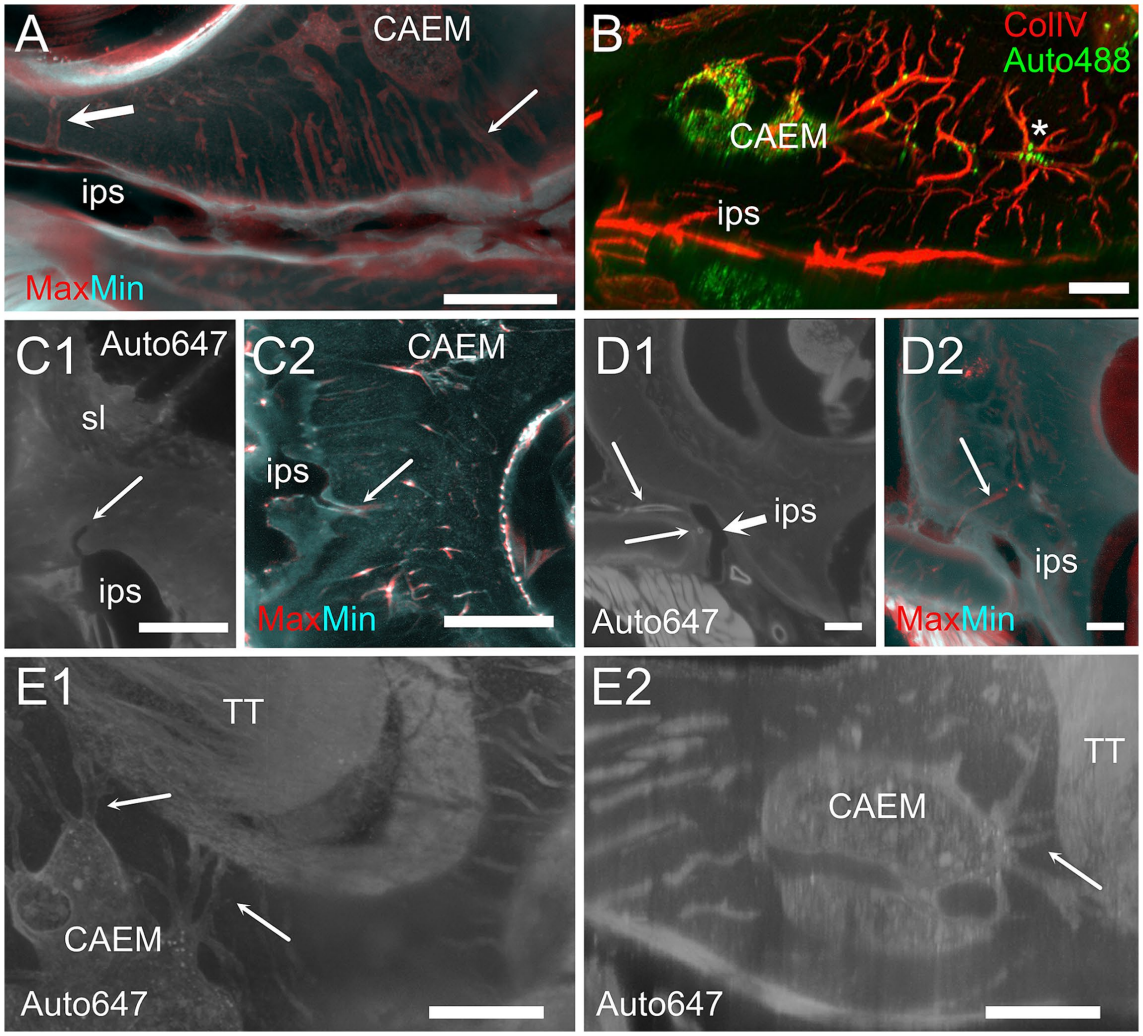
Extracochlear connections of CAEM. **(A)** Maxmin projection from 250 μm stacks from 647 nm autofluorescence signal. Arrows show points of confluence of channels into the perisinusal space (large arrow) or sinus (small arrow). **(B)** Vascular CAEM connections to the inferior petrosal sinus. Asterisk labels reticular marrow. **(C1)** MinIP from a 90 μm stack showing the course of a channel draining in the petrosal sinus (arrow). The canaliculi arrived in the vicinity of the spiral ligament. **(C2)** Maxmin projection from 100 μm stacks from ColIV signal. Arrow shows confluence of channels into the petrosal sinus. **(D1)** Optical section showing the course of thick-wall vessels (thin arrows) running parallel to the petrosal sinus wall (thick arrow). **(D2)** Maxmin projection from 100 μm stacks from 647 nm autofluorescence signal. The arrow shows the continuation of the thick wall vessel in **(D1)** up to CAEM. **(E)** Bone channels connecting CAEM to the tensor tympani fossa (arrows). **(E1)** MIP of 150 μm stack, horizontal; **(E2)** MIP of 30 μm stack, resliced on the vertical. Image in panel **(A,E1,E2)** are from R3, **(B,C2)** from R15b, **(C1)** from R1a, **(D1,D2)** from R8b. Scale bars are 500 μm in **(A,C2,D)**, 200 μm in **(B,E)** and 300 μm in **(C1)**.

CAEM (Figure 5) was an isolated marrow cluster with few small spheroid lobules confined to the apicomedial side of the cochlea (Figure 5A; see also Figures 1, 2). CAEM volume was significantly larger in females than males (see Figure 3D). In the 647 nm autofluorescence signal, thin bone channels were visible connecting CAEM to the cochlear spiral ligament of the second turn; when followed in their entirety (Figure 5B), these channels showed looping pathways starting from the marrow, reaching the spiral ligament and coasting it, and then returning to the marrow. Similar loops were seen with ColIV labeling (Figure 5C), suggesting at least some of the loops are vascular. Vascular systems of the cochlea and surrounding bone always appeared separated (Figure 5D). However, bone channel systems were not (Figure 6). The inferior petrosal sinus was connected to CAEM through straight bone channels (Figure 6A) and blood vessels (Figure 6B) but was also connected to the spiral ligament border (Figure 6A). Vascular channels extending to the dural sinus could reach into the sinus lumen (therefore representing venules, Figure 6C) or connect to small vessels within the connective tissue surrounding the sinus (Figure 6D), which could represent arterioles or lymphatic collectors. In addition to the dural sinus, CAEM was unexpectedly also connected through several channels to the tensor tympani fossa of the middle ear (Figure 6E), in all samples where both structures were clearly visible (*n* = 16). Channels were measured in high resolution image stacks from 647 nm autofluorescence or TO-PRO, giving an average number of 6 ± 2 connections (*n* = 9) with an average diameter of 19.0 ± 19.9 μm, not significantly different (*p* = 0.1) from the diameter of small channels connecting bone marrow and dural sinuses. In one sample, it was possible to follow ColIV+ blood vessels in these channels. These connections could allow inflammatory mediators and cells to be exchanged between the cochlea and the tensor tympani muscle.

### Cochlear base

**Figure 7:**
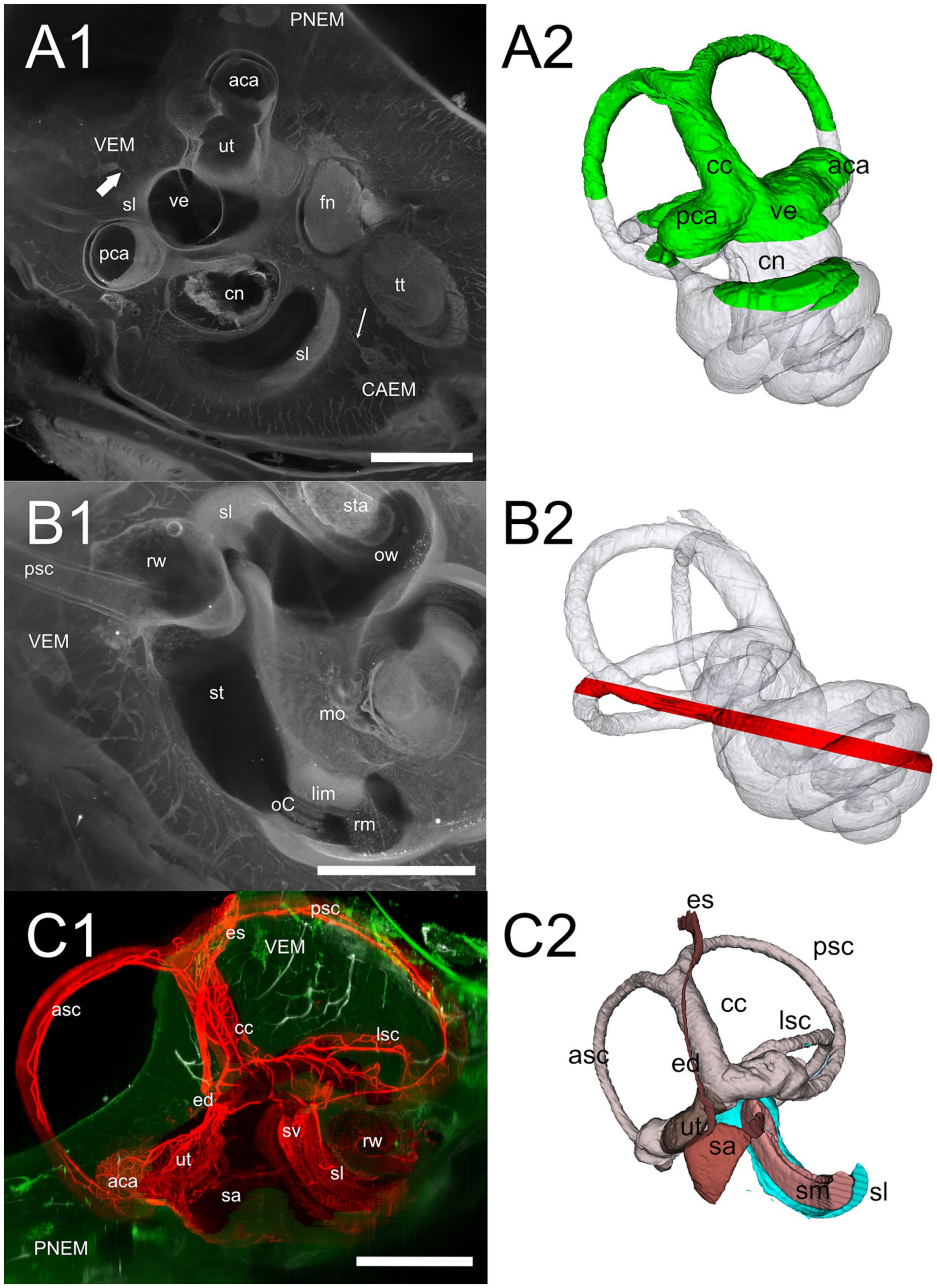
Cochlear base. **(A1)** 647 nm autofluorescence signal from a single optical section through the cochlear base showing most of the petrosal bone. All three marrow clusters are visible: CAEM in association with the apicomedial cochlea, PNEM in association with the anterior canal, and VEM in association with the basal cochlea. Bone marrow connections are visible from CAEM to spiral ligament of the second turn (thin arrow) and from VEM to spiral ligament of the cochlear hook (thick arrow). **(A2)** Whole bone labyrinth 3D reconstruction showing the sectioning plane in **(A1)**. Green indicates the volume over the optical section, gray the volume under it. **(B1)** MIP from a 200 μm stack starting 520 μm more lateral to the section in **(A1)**, showing bone marrow connections to the scala tympani periosteum and round window. **(B2)** Whole bone labyrinth 3D reconstruction showing in red the position and z-depth of the stack used for B1 MIP. **(C1)** Vascular connections at the cochlear base, showing the terminal segment of spiral ligament and stria vascularis. Red: MIP of a 2.5 mm stack showing membranous labyrinth vessels (ColIV signal). White: MIP of a 200 μm stack showing temporal bone vessels (ColIV signal). Green, MIP of a 200 μm stack showing bone marrow (488 nm autofluorescence signal). **(C2)** 3D reconstruction of the membranous labyrinth, with similar orientation and sectioning as panel **(C1)**. Images in panel **(A1,B1)** are from sample R3, image in **(C1)** from R15b. All 3D reconstructions are from sample R3. All scale bars: 1 mm.

At the cochlear base (Figure 7) bone channels were more complex, showing a very interconnected network (Figure 7A). Whereas CAEM was associated to the cochlear apex only, a separate reticular or mixed reticular-spheroid bone marrow cluster reached the hook spiral ligament (Figure 7A) and scala tympani periosteum (Figure 7B). Vascular labeling with ColIV (Figure 7C) revealed an asymmetry in bone microvascular density at the cochlear base, with higher density around the crus commune up to the round window, and lower density toward the cochlear modiolus and otolithic maculae. Bone marrow was only seen in the regions of high vascular density, which in the rat correspond to endochondral bone (41).

**Figure 8:**
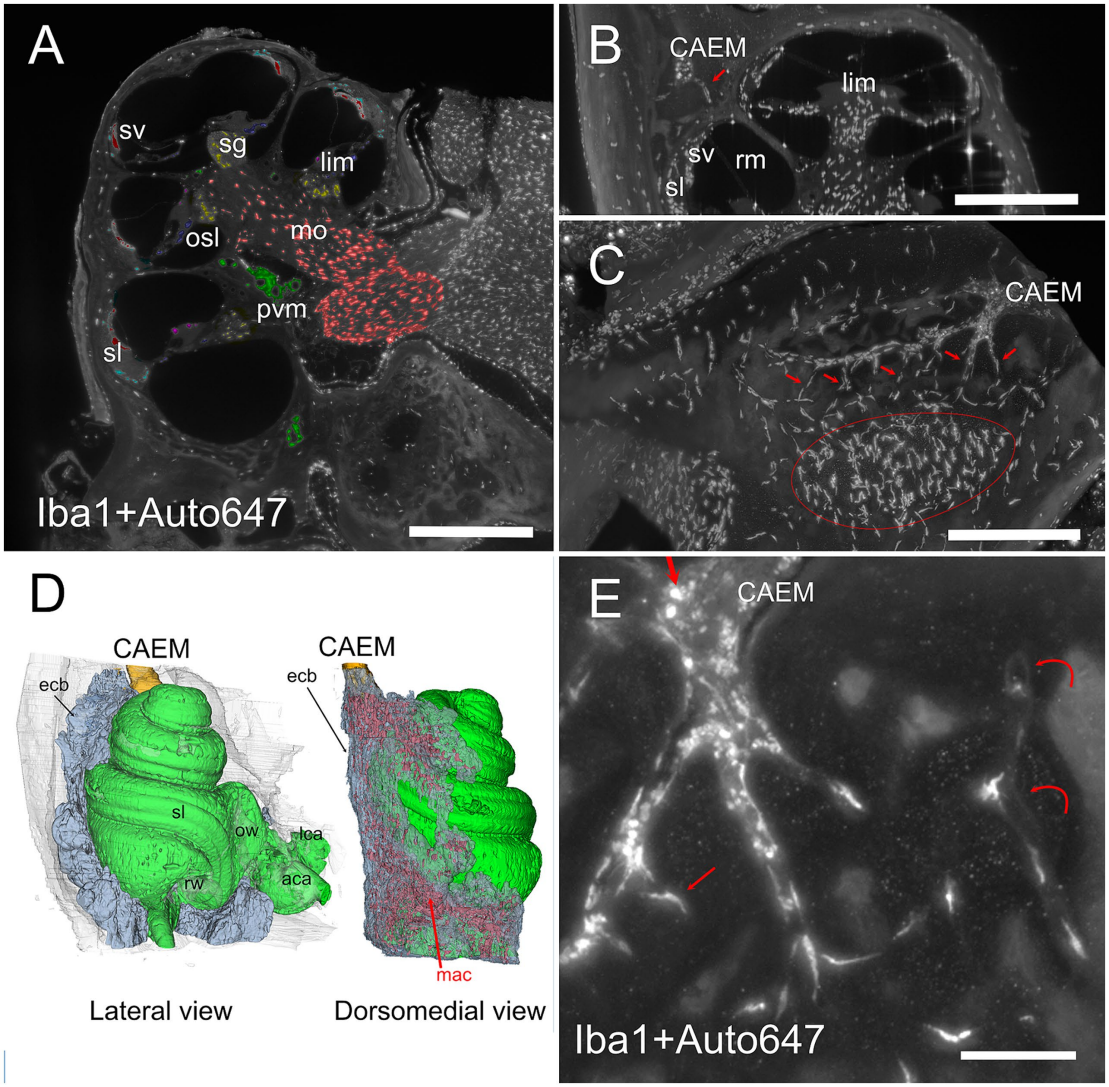
Bone marrow-related macrophages. **(A)** Iba1 labeling of the cochlea, displaying (with pseudocolors) the known macrophage populations from the literature in the spiral ganglion (yellow), osseous spiral lamina (blue), limbus (purple), ligament (cyan), stria vascularis (red), perivascular (green), and modiolar (orange). **(B)** MIP from 30 μm stack of a section of the cochlea showing a connection between CAEM and spiral ligament (arrow) lined by macrophages. **(C)** MIP from 100 μm stack of the region from CAEMto the spiral ligament border (outlined in red). Connections are lined by macrophages (arrows). **(D)** 3D reconstruction of the endochondral bone (semitransparent) around the cochlea showing its high macrophage density (in red). CAEM is partially shown to see its relative position. **(E)** High magnification of a CAEM cluster showing bone marrow macrophages (thick straight arrows) and bone macrophages (thin arrows). MIP of 10 μm stack, Iba1 plus autofluorescence signal at 647. Curved arrows show channel borders visible in the autofluorescence signal. Images in panels **(A,C,D,E)** are from sample R1a, image in **(B)** from R1b. Scale bars: **(A,B)** 500 μm; **(C)** 250 μm; **(E)** 100 μm.

All macrophage populations previously described in the literature (42) could be identified in the cochlea by Iba1 labeling (Figure 8A). In addition, Iba1+ cells were seen within bone marrow and bone channels connecting CAEM to the spiral ligament (Figures 8B,C) and in endochondral bone at the cochlear base (Figure 8D; see also Figure 1E). At higher magnification, Iba1+ cells in bone marrow and channels displayed different shapes (Figure 8E); marrow Iba1+ cells were rounded, whereas those associated with channels were elongated. Although both Iba1 and autofluorescence signals were excited at 647 nm, Iba1 labeling was brighter than autofluorescence, and displayed distribution patterns which were never observed with 647 nm autofluorescence only (compare Figure 8E with Figures 4A,G,H).

### Petrosal non-endochondral bone marrow (PNEM)

**Figure 9:**
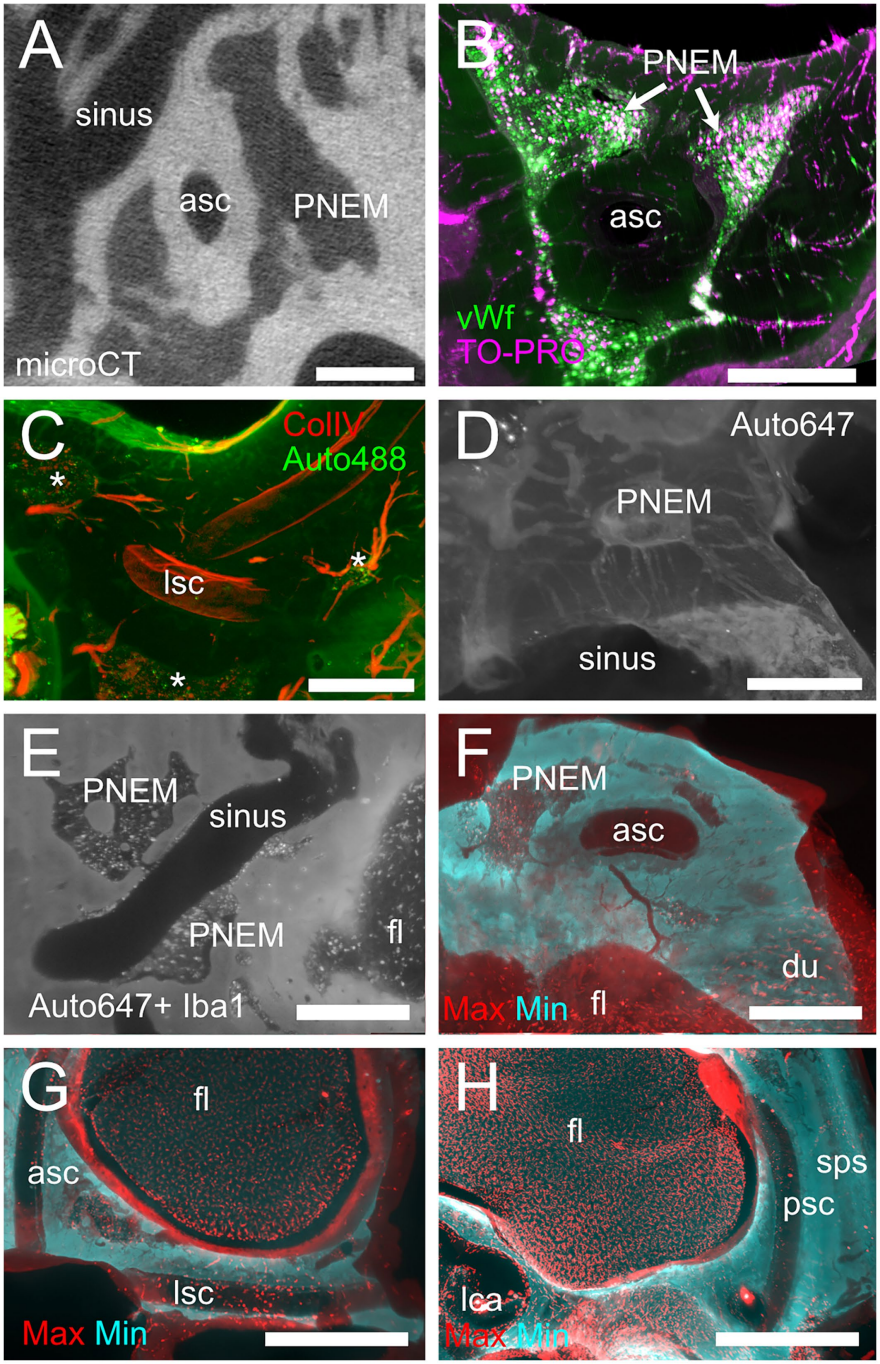
Petrosal non-endochondral marrow (PNEM). **(A)** Single retroprojected section from microCT of the anterior canal simple arm. **(B)** MIP from a 100 μm stack from the anterior semicircular canal, labeled for vWf (green) and TO-PRO (magenta). Large PNEM marrow lobules are visible in both **(A,B)**. **(C)** PNEM blood vessels penetrating the bony labyrinth around canal arms. MIP from a 100 μm stack from the crossing of lateral and posterior semicircular canals, showing autofluorescence at 488 nm (green) and ColIV (red). Asterisks indicate marrow lobules. **(D)** PNEM vascular connections to the superior petrosal dural sinus. MIP from a 200 μm stack showing autofluorescence at 647 nm. **(E)** Direct opening of PNEM into the petrosal sinus lumen. Single optical section, Iba1+ 647 nm autofluorescence. **(F)** Maxmin projection from 500 μm stacks from 647 Iba1 signal. Note the presence of labeled cells in bone marrow, in parallel channels in the dura, and in the cerebellar flocculus (microglia). Bone channels from PNEM do not contain Iba1+ cells. **(G)** Maxmin projection from 300 μm stacks from 647 Iba1 signal showing macrophage population associated with the anterior and lateral canal. **(H)** Maxmin projection from 300 μm stacks from 647 Iba1 signal showing macrophage population associated with the posterior canal. Image in panel **(A)** is from sample R16a, **(B)** from R11a, **(C)** from R15b, **(D)** from R3, **(E–H)** from R1a. Scale bars: **(A–F)**: 500 μm; **(G,H)**: 1 mm.

Petrosal non-endochondral bone marrow (PNEM) had a more complex geometry than endochondral bone marrow, following the semicircular canal arms and extending into the crista petrosa (Figure 9; see also Figures 1, 2). Bone marrow in the crista petrosa could be continuous with that surrounding semicircular canals (*n* = 6 samples) or form a separate island (*n* = 5 samples; see also Figure 3B). Inhibition of bone remodeling was clearly visible when observing perpendicular sections around simple canal arms (Figures 9A,B) where the latter were surrounded by a circle of dense bone with sparse thin vascular channels connecting them to the surrounding marrow. Bone marrow vessels could penetrate the bony labyrinth through these channels, and reach the canal periosteum, but the vascular network of membranous canals appeared separate (Figure 9C). PNEM was extensively connected to the superior petrosal dural sinus through channels similar to those found for CAEM in the inferior petrosal sinus (Figure 9D). In addition, most commonly around the crus commune confluence, bone marrow opened directly into the dural sinus, with a large junction where marrow cells appeared to be exposed to the sinus lumen (Figure 9E).

The distribution of Iba1+ cells in PNEM was very different from that of endochondral bone: although round Iba1+ cells were present within bone marrow, little or no elongated Iba1+ cells were seen following bone channels (Figure 9F). Semicircular canal periosteum, perilymph, and periosteal bone channels displayed Iba1+ cells, with the ampullae and lateral canal showing higher densities than anterior and posterior canal arms (Figures 9G,H). At the crus commune, endochondral bone was present, and therefore PNEM was replaced by VEM; in most samples, the two clusters were separated, but in two samples they were continuous, gradually changing properties.

### Vestibular endochondral bone marrow (VEM)

**Figure 10:**
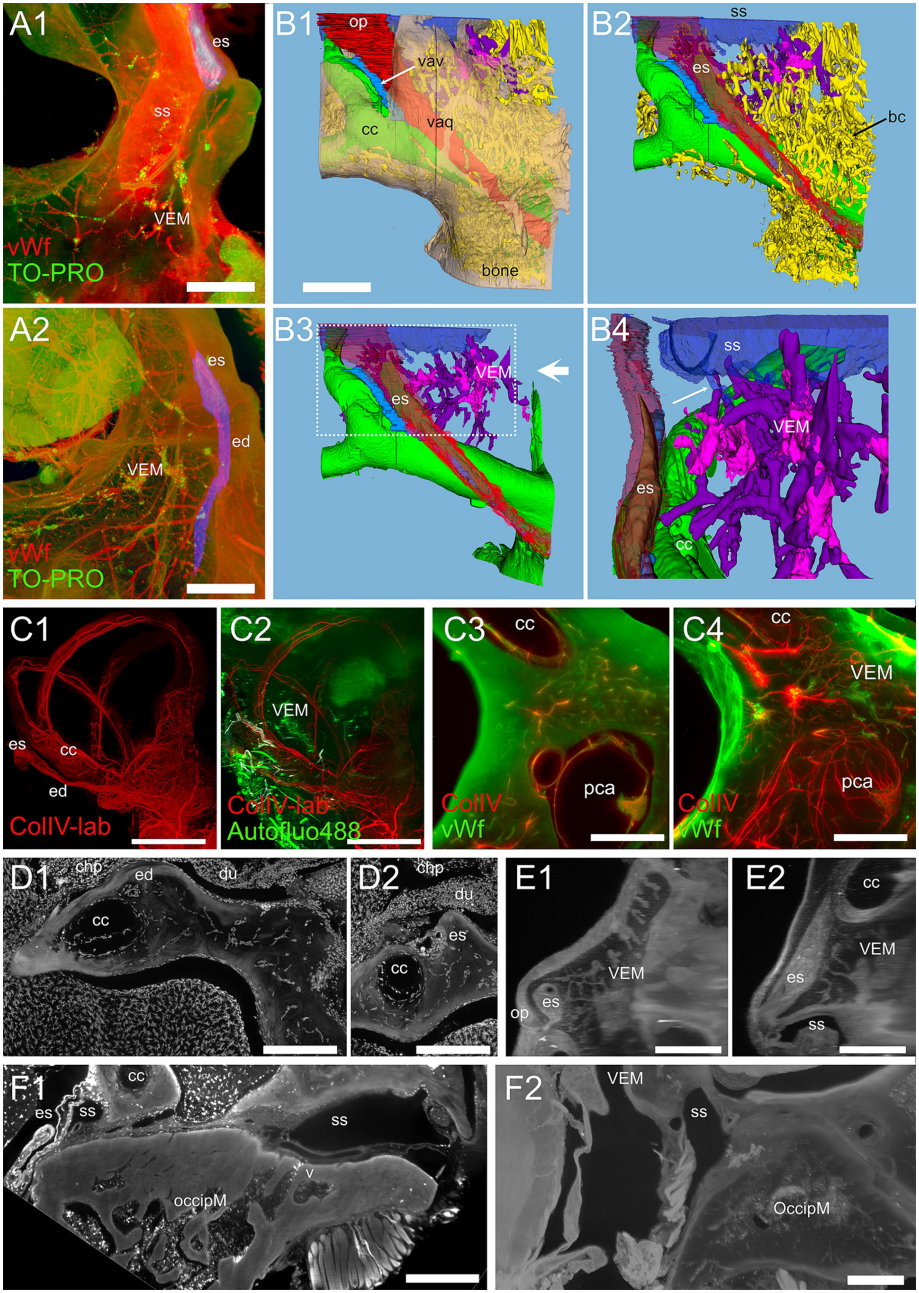
Vestibular endochondral bone marrow (VEM). **(A)** VEM network around the endolymphatic sac and duct. **(A1)** MIP of 360 μm stack at the level of endolymphatic sac. **(A2)** MIP of 1.5 mm stack at the level of the endolymphatic duct. Red: vWf; green: TO-PRO; blue: segmented vestibular aqueduct. **(B)** 3D reconstruction from segmentations of the stack in **(A)**. **(B1)** Vestibular aqueduct (red) is visible through semitransparent bone (beige) until the operculum. Arrow indicates the vestibular aqueduct vein. **(B2)** Removal of bone and semitransparent vestibular aqueduct shows a large number of bone channels (arrow) wrapping the endolymphatic duct and sac and the crus commune. **(B3)** VEM and its directly attached channels are shown in purple after removal of other bone channels. Large arrow indicate point of view of **(B4)**. **(B4)** Magnification of B3 inset, rotated on the vertical axis to show relations to the endolymphatic sac. Channels attach to the sigmoid sinus (arrow) and are well evident around the proximal part of the endolymphatic sac. **(C)** VEM vascular channels. **(C1)** MIP of a 2.7 mm stack of ColIV signal, after segmentation of the labyrinth; **(C2)** same MIP as **(C1)** with the addition of a MIP of an 800 μm stack of autofluorescence signals (green) showing VEM location; **(C3)** single optical slice of ColIV (red) and vWf (green) signals showing VEM at the beginning of the crus commune; **(C4)** MIP of a 430 μm stack around the section in **(C3)**. **(D)** VEM-associated macrophages. MIPs of a 90 μm stack at the level of the distal duct **(D1)** and proximal sac **(D2)**. VEM was not visible in the stack but connected to the channels in deeper sections. **(E)** MIPs of a 200 μm stack of 647 nm autofluorescence signal showing VEM and channels connecting it to the proximal sac. E1 and E2 are orthogonal. **(F)** Occipital bone marrow. **(F1)** MIP of a 100 μm stack of Iba1 signal showing a diploic vein draining from occipital bone marrow into the sigmoid sinus. **(F2)** MIP of a 200 μm stack of 647 nm autofluorescence signal showing thin bone channels connecting occipital bone marrow with the sigmoid sinus. Image in panels **(A,B)** are from sample R11a, **(C1,C2)** from 15b, **(C3,C4)** from R15a, **(D)** from R1b, **(E)** from R3, F1 from R1a, **(F2)** from R8b. All scale bars 500 μm except **(C1,C2)**: 1 mm.

Vestibular endochondral bone marrow (Figure 10) displayed a small volume, and its lobules were variable in position, size and shape (see Figures 2, 3): in 5 samples it was reticular (as in Figures 4F,I), in 6 samples it was spheroidal, and in 3 mixed (i.e., contained both small reticular and larger spheroidal lobules); in 2 samples, it was continuous with PNEM so the nature of its lobules was less clear. Reticular marrow was difficult to delineate unless cell distribution was observed with 647 nm autofluorescence, TO-PRO, or vWf; therefore, it is very likely that in microCT observations the contribution of reticular marrow is underestimated. High resolution vWf and TO-PRO labeling (Figure 10A) allowed us to reconstruct the endolymphatic duct and sac together with the vestibular aqueduct vein and sigmoid sinus (Figure 10B), obtaining a similar connectivity as described for brain-associated bone marrow (43), in spite of the small size of marrow lobules. Bone marrow channels around the endolymphatic duct and sac were extremely ramified (Figure 10B), mostly vascular (Figure 10C), and macrophage-rich (Figure 10D), forming an interconnected network reaching the endolymphatic sac, bone surface, crus commune periosteum and sigmoid sinus (Figure 10E). Although not part of the petrosal bone, occipital bone marrow was connected to the sigmoid sinus through diploic veins and thin channels (Figure 10F).

### Ectotympanic bone marrow

**Figure 11:**
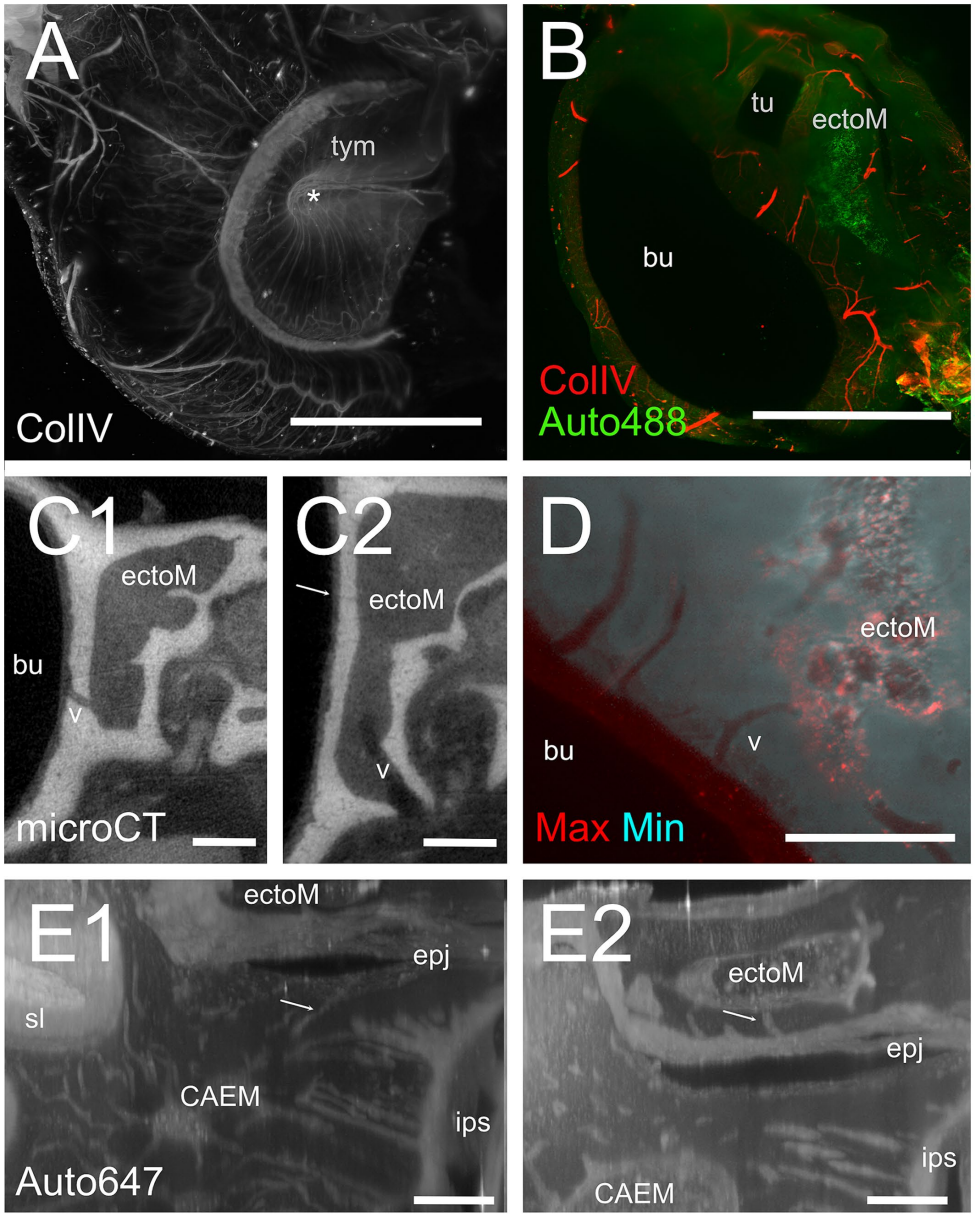
Ectotympanic bone marrow. **(A)** Middle ear vasculature showing the tympanic membrane. MIP of a 5 mm stack of ColIV labeling. Asterisk shows the tympanic umbo. **(B)** MIP of a 250 μm stack of 488 nm autofluorescence and ColIV signal showing the ectotympanic bone marrow cluster associated to tubal ostium and its vascular connections to the bulla. **(C)** microCT section showing a diploic vessel channel **(C1)** and thinner bone channels **(C2)** connecting the ectotympanic bone marrow with the bulla. Arrow indicates a thin bone channel. **(D)** Maxmin projection of a 100 μm stack showing bone channels from ectotympanic bone marrow to the bulla inner surface. 488 nm autofluorescence. **(E)** MIP of 30 μm stacks showing ectotympanic bone marrow **(E1)** and CAEM **(E2)** connections (arrows) to the ectotympanic-petrosal junction. Connections from ectotympanic bone marrow and CAEM were not visible in the same stack, but the regions were adjacent. Image in panels **(A,B,D)** are from sample R15b, **(C1,C2)** from 16b, **(E)** from R3. Scale bars: **(A,B)** 2 mm; **(C,D)** 500 μm; **(E)** 200 μm.

Although the rat middle ear is surrounded by an ectotympanic bone, which is separated from the petrosal bone (18), it appears important to compare middle ear- and inner ear-associated bone marrow features and distribution, since the middle ear represents the principal site of entry of pathogens and drugs for the inner ear (44, 45). Ectotympanic bone marrow (Figure 11) was found in two large islands (total volume 1.54 ± 0.79 mm^3^, *n* = 4) surrounding the tubal and tympanic opening (see Figure 2C). The bulla was vascularized by parallel vessels reaching the mucosa (Figure 11A), which passed through bone marrow clusters (Figure 11B). Both large and small bone channels were visible connecting ectotympanic bone marrow to the middle ear mucosa with microCT (Figure 11C). In lightsheet scans, large channels were visible in 488 autofluorescence, but unfortunately, for the samples where the whole bulla was available, 647 signal was unavailable. On the other hand, the ectotympanic marrow closer to the petrosal bone was available in most samples, and thin channels were visible toward the suture between ectotympanic and petrosal bone (Figure 11E), in a position right opposite to CAEM.

## Discussion

In this work we characterized bone marrow distribution in the rat temporal bone and its connections with inner ear structures, by analyzing microCT and lightsheet image stacks of immunolabeled cleared temporal bones; whereas the former technique is faster and easier (and allows *in vivo* recordings), the latter allowed us to identify bone cavities as bone marrow, thanks to the labeling of immune cell types which are localized in hematopoietic niches, such as megakaryocytes (14). The localization of megakaryocytes in cleared bone marrow has been shown in other bones to reflect the distribution of stem cells (46). In addition, lightsheet imaging allowed us to distinguish features in the decalcified bone matrix which delineated petrosal endochondral bone (38, 40), and therefore allowed us to observe endochondral bone marrow clusters. The size and perivascular location of endochondral bone marrow lobules justifies their neglect in classical temporal bone histology: without selective labeling, most immune cells cannot be identified, the small size of marrow clusters hinders their analysis with conventional bone marrow tools, such as needle aspiration; on the other hand, since bone marrow cells are only loosely attached to each other (47), slice histology may lead to marrow cell detachment and loss, whereas tissue clearing of intact bones has been shown to give more unbiased cell density estimates (14). To our knowledge, this is the first complete reconstruction of temporal bone marrow distribution in rodents.

In the brain, calvarial bone marrow is connected to the dura and CSF spaces through bone microvascular channels (43) which allow passage of immune cells, pathogens, and CSF signals triggering bone marrow cell release (2), supporting a major role of local bone marrow in neuroimmune communication. In particular, the calvarial–meningeal path allows transfer to the dura of unique myeloid cells (48), and of immature B lymphocytes (49). In the temporal bone, we found that marrow clusters were connected to the dura through channels of similar diameter as calvarial marrow (2), whereas connections to the inner ear appeared to coast the perilymphatic compartment rather than opening into it. It is important to consider that several regions of the otic capsule bone are porous (50), and that bone channels around the inner ear have been seen to fill with markers upon fluid injection in the inner ear. In particular, injection of a marker in the cochlear perilymph was found to label bony channels outside the spiral ligament (51) and large endolymph injections in the scala media to label bony channels outside the endolymphatic duct (52). The latter have been suggested to form a “valve-like” system in the regulation of inner ear pressures (53). The distribution of bone marrow found in the present work suggests that, upon expansion of inner ear fluids, bone channels would fill through an extracellular matrix “sieve” and feed to local bone marrow clusters, which would therefore be exposed to inner ear antigens. This would represent an optimal mechanism to only expose local immune cells to inner ear contents in the presence of an inflammatory response.

In calvarial bones, the channel architecture (maintained by osteoclasts) contains specialized microvessels traversing both cortical bone plates and bone marrow (54). The temporal bone otic capsule, however, displays nuclei of endochondral ossification similar to long bones rather than membranous ossification as in calvarial bones (55) and osteoprotegerin secreted by the membranous labyrinth (51) blocks the signaling involved in the differentiation of osteoclasts (56). It is therefore not surprising to find that temporal bone marrow architecture is more complex than in calvarial bones, with marrow clusters of different size and connectivity. Bone matrix autofluorescence intensity at 647 nm allowed delineation of the otic capsule endochondral bone characterized by an interconnected network of macrophage-rich vascular channels connected to reticular bone marrow clusters containing Iba1+ cells and megakaryocytes. The architecture of rat endochondral bone we observed fits with what was characterized in previous studies, where it was found to be very vascularized and enriched in globuli ossei rather than interglobular substance (40). Although the techniques used in these previous studies did not allow labeling of selective immune cell populations nor 3D reconstruction, and therefore bone marrow was not investigated in these studies, comparative measurements of bone properties from these studies suggest that the rat would be the optimal model to study endochondral bone marrow, since endochondral bone vascularization is much higher in rat than in most other animal models (41). Given that the links between bone marrow, bone metabolism, and inflammation are being elucidated (57), studying endochondral bone marrow may be crucial in understanding inner ear pathologies that link inflammation to aberrant vascular and bone changes, such as Menière’s disease (58, 59) or otosclerosis (60).

Macrophages represent an important bone cell population (61) and have been observed in large quantities in pathological human otic capsule (62); bone marrow macrophages have been observed in calvarial bone marrow (63) and appear involved in hematopoiesis (64). Although several studies have focused on macrophage populations in the inner ear (see (1, 42); PUBMED search for “macrophages AND (“inner ear” OR cochlea)” retrieved 338 papers as the time of submission of the present work), the populations residing in bone marrow and in its connections, which may be involved in bone marrow plasticity (4), are still uncharacterized. The observed megakaryocytes could also be related to this plasticity, since these cells have been found to secrete osteoprotegerin and regulate bone marrow remodeling (65, 66).

The presence in the temporal bone of three marrow clusters with different connections and geometrical features raises issues regarding their possible different roles. Of the observed marrow clusters, CAEM appears to be in the optimal position to produce cells reaching the cochlea through the spiral ligament. Within the cochlea, nonresident immune cells have been found to enter the perilymph through two different microvascular routes, i.e., crossing the spiral ligament and the modiolus (1). Experiments have shown that innate immune cells enter the perilymph through the spiral ligament (67) whereas lymphocytes enter through modiolar venules (68). Moreover, lateral wall macrophages (and a subpopulation of spiral ligament fibrocytes) derive from bone marrow cells (69–71) and the proportion of bone marrow-derived macrophages increases with age (72). Our data would support a local bone marrow production of innate cells reaching the lateral wall, as in the brain (48), since both CAEM and VEM are connected to the spiral ligament and external cochlear wall but not to the cochlear modiolus. The presence of separate clusters connecting to the apical and basal parts of the cochlea appears interesting, considering that the two parts display different weakness to diseases (73). Similarly, the different CAEM volume between male and female animals has to be considered among the factors differentially affecting hearing between genders (74).

Although other immune cell types have been found in the cochlea (75) especially upon inflammation (76), in the labyrinth non-macrophage immune cells appear largely confined to the endolymphatic sac (77), the terminal expansion of the endolymphatic duct, which is surrounded by tortuous bone channels (78, 79), some of which connect to bone marrow (4). It is therefore possible that VEM contains different cell populations, more biased toward adaptive immunity. It must be emphasized that the small size (less than 0.1 microliter) and percentage on total temporal marrow (about 3%) of VEM would make its analysis difficult with non-spatial approaches, such as FACS or single-cell transcriptome, where VEM features would be lost by mixing with the remaining marrow. Further experiments will compare rat marrow distribution with mouse, where single cell transcriptome datasets for temporal bone immune cells (76) and more complete marker sets for bone marrow niches are available.

Although rodent temporal bone displays several important differences from human temporal bone (separate ectotympanic bone, deep subarcuate fossa containing the paraflocculus and surrounded by semicircular canals, persistent stapedial artery, lack of pneumatization), and although the immune system is much more species-specific (80) than the inner ear (81), the characterization of rodent bone marrow will help the understanding of human temporal bone marrow. Since the distribution of temporal bone marrow in human is heterogeneous (6), and since functional experiments can be performed in animal models, comparing the temporal bone marrow distribution and composition among the main animal models used for the inner ear (guinea pig, mouse, rat, large animals such as pig) and with human temporal bone marrow appears important to elucidate the mechanisms of inner ear immune responses. From our data, the location of CAEM appears analogous to that of petrous apex marrow in humans, which is also similarly exposed to middle ear-derived pathogens (73). The much simpler structure of the rat CAEM in respect to the human petrosal marrow would make this animal a good model for studying the local immune responses during infection spread from middle to inner ear, since it appears that the ectotympanic-petrosal suture is the only site of communication between middle and inner ear bone marrow. Middle ear-associated marrow is located in the ectotympanic bone (bulla) at sites of contact to the outer environment: two ring-shaped clusters are found around the tubal orifice and the external auditory meatus. Middle ear bone marrow has been found to provide local immune protection in otitis in mice (3) and is connected to the middle ear mucosa through bone channels. We have seen similar channels in rat, connecting ectotympanic bone marrow to the middle ear cavity. In addition, the same marrow forms connections at the border between ectotympanic and petrosal bone, facing CAEM. At this region it is likely that antigens, immune cells and cytokines from the two compartments interact, alerting inner ear immune defenses of a middle ear infection and limiting its spread. Since we also saw connections between CAEM and PNEM and brain dura, it appears likely that CAEM and PNEM clusters draw a “border” around the inner ear structures, coordinating the immune responses of the middle ear, inner ear, and brain. The stronger connections of CAEM and VEM to the inner ear when compared to PNEM, where bone channels toward the labyrinth are scarce, may reflect the different vulnerability of the vestibular system with respect to the cochlea. Optimization of immune protection in the vestibular system is suggested by the macrophage distribution in canal arms: the lateral canal, which is closer to the bone surface and therefore potentially more exposed to pathogens or toxic substances, displays a larger pool of macrophages than the other canal arms, which run deeper.

The peculiar connections of CAEM to the tensor tympani fossa, on the other hand, are a very interesting separate issue, because they would provide the route that was hypothesized for inflammatory signals from the cochlea to the tensor tympani in the acoustic shock model linked to tinnitus, hyperacusis, and ear fullness (82). The tensor tympani muscle, connecting the malleus to the Eustachian tube (83), can be activated by both auditory and non-auditory stimuli, such as tactile stimulation of the external canal, movements of the jaw, and even acute or chronic stress (84). In the acoustic shock model, noise trauma to the cochlea occurring together with additional factors activating the tensor tympani could induce inflammation in the muscle, triggering a positive feedback cycle that would induce muscle damage similarly to what has been found in myofascial trigger point, involving the release of inflammatory molecules, mast cell degranulation, and pain (79). Our finding that CAEM is connected to the tensor tympani would suggest that inflammation in the muscle could also affect cochlear response, and damage or inflammation in the cochlea could directly affect the tensor tympani. Given that tensor tympani anomalous responses have been associated with tinnitus (85, 86), which is also affected by inflammation (87), it would be interesting to study inflammatory responses around CAEM in a tinnitus model.

This work gave an overview of the rat temporal bone marrow distribution and connectivity, which yields important context for functional studies of inner ear immunity in animal models. Limitations of this study are the limited number of samples and markers employed and the lack of functional experiments, which leave to speculation the functional roles of marrow clusters. The importance of the present results, however, lies in the recognition of separate marrow clusters showing different features and “private connectivity” for different regions of the inner ear, and in the quantification of volumes, showing that the cluster associated to the regions most involved in the inner ear immune responses represents only a small fraction of the total temporal bone marrow.

## Data availability statement

The raw data supporting the conclusions of this article will be made available by the authors, without undue reservation.

## Ethics statement

The animal study was approved by Italian Ministry of Health and University of Pavia Animal Welfare Office (OPBA). The study was conducted in accordance with the local legislation and institutional requirements.

## Author contributions

PP: Conceptualization, Data curation, Formal analysis, Funding acquisition, Investigation, Methodology, Project administration, Resources, Software, Supervision, Validation, Visualization, Writing – original draft, Writing – review & editing. DC: Investigation, Methodology, Software, Visualization, Writing – original draft, Writing – review & editing. EV: Data curation, Formal analysis, Software, Visualization, Writing – original draft, Writing – review & editing. LB: Investigation, Visualization, Writing – original draft, Writing – review & editing. IG: Investigation, Visualization, Writing – original draft, Writing – review & editing. FV: Investigation, Methodology, Writing – original draft, Writing – review & editing. RP: Writing – original draft, Writing – review & editing.

## Glossary

Aca, lca, pca: Anterior, lateral, posterior semicircular canal ampulla
asc, lsc, psc: Anterior, lateral, posterior semicircular canals
bc: Bone channels
VEM: Vestibular endochondral marrow
BM: Bone marrow
bsb: Basisphenoid bone
bst: Brainstem
bu: Bulla
CAEM: Cochlear apex endochondral marrow
caq: Cochlear aqueduct
cc: Crus commune
cn: Cochlear nerve
co: Cochlea
conu: Cochlear nuclei
cp: Crista petrosa
chp: Choroid plexus
dr: Ductus reuniens
ds: Ductus sacculi
du: Dura
eam: External auditory meatus
ecb: Endochondral bone
ectoM: Ectotympanic bone marrow
ed: Endolymphatic duct
eob: Endosteal bone
epj: Ectotympanic-petrosal junction
es: Endolymphatic sac
fc: Facial canal
fl: Floccule
fn: Facial nerve
hp: Habenula perforata
ijv: Internal jugular vein
ips: Inferior petrosal sinus
lam: Lamina
lim: Spiral limbus
mac: Macrophage
MIP: Maximum intensity projection
MinIP: Minimum intensity projection
mo: Modiolus
PNEM: petrosal non-endochondral marrow
ob: Occipital bone
oc: Otic capsule
oC: Organ of Corti
occipM: Occipital bone marrow
op: Operculum
osl: Osseous spiral lamina
ow: Oval window
pgv: Postglenoid vein
pvm: Perivascular macrophages
ra: Radial arteries
rem: Recessus meatus
rm: Reissner’s membrane
rw: Round window
sa: Saccule
saf: Subarcuate fossa
sca: Scarpa’s ganglion
sct: Spiral cribriform tract
sg: Spiral ganglion
sl: Spiral ligament
sla: Spiral lamina
sm: Scala media
sps: Superior petrosal sinus
ss: Sigmoid sinus
st: Scala tympani
sta: Stapes
stm: Stapedial muscle
sv: Stria vascularis
sva: Superior vestibular area
ts: Transverse sinus
tt: Tensor tympani
tu: Tubal canal
tym: Tympanum
ut: Utricle
v: Vein
vaq: Vestibular aqueduct
vav: Vestibular aqueduct vein
ve: Vestibule
vv: Vertebral vein
