# Supplementary material for: Temporal bone marrow of the rat and its connections to the inner ear
Source: Front Neurol. 2024 May 16;15:1386654. doi: 10.3389/fneur.2024.1386654 (PMC11137668; doi:10.3389/fneur.2024.1386654)
Supplement: Supplementary file 1 [file Presentation_1.zip › Supplementary Materials Captions.docx]

Temporal bone marrow of the rat and its connections to the inner ear

Paola Perin, Daniele Cossellu, Elisa Vivado, Laura Batti, Ivana Gantar, Fabian Voigt, Roberto Pizzala

*** Correspondence:** Corresponding Author: paola.perin@unipv.it

**Supplementary material**

**Supplementary Figure 1.** MaxMin projection. A: maximum intensity projection over 300 µm z-depth of the sample depicted in Figure 8G, false-colored red; B: minimum intensity projection over 300 µm z-depth of the sample depicted in Figure 9G, false-colored cyan; C: maxmin projection image obtained by combining images from A and B.

**Supplementary Figure 2.** 3D reconstruction of interparietal bone (part of calvaria) and marrow cavity from R16. Bone is semitransparent. Green: bone marrow cavity. Note the presence of a single diploic marrow island, similar to Figure 2D in Kolabas et al. 2023. Scale bar: 2 mm.

**Supplementary Video.** 3D reconstruction of temporal bone structures from microCT scans of sample R16b. In order of appearance: bony labyrinth (green); cochlear apex marrow (orange); basal enchondral bone marrow (purple); non-enchondral petrosal bone marrow (cyan); ectotympanic bone marrow (pink); petrosal bone (semitransparent); occipital bone marrow (blue).
