# Supplementary figures and images for: Temporal bone marrow of the rat and its connections to the inner ear
Source: Front Neurol. 2024 May 16;15:1386654. doi: 10.3389/fneur.2024.1386654 (PMC11137668; doi:10.3389/fneur.2024.1386654)

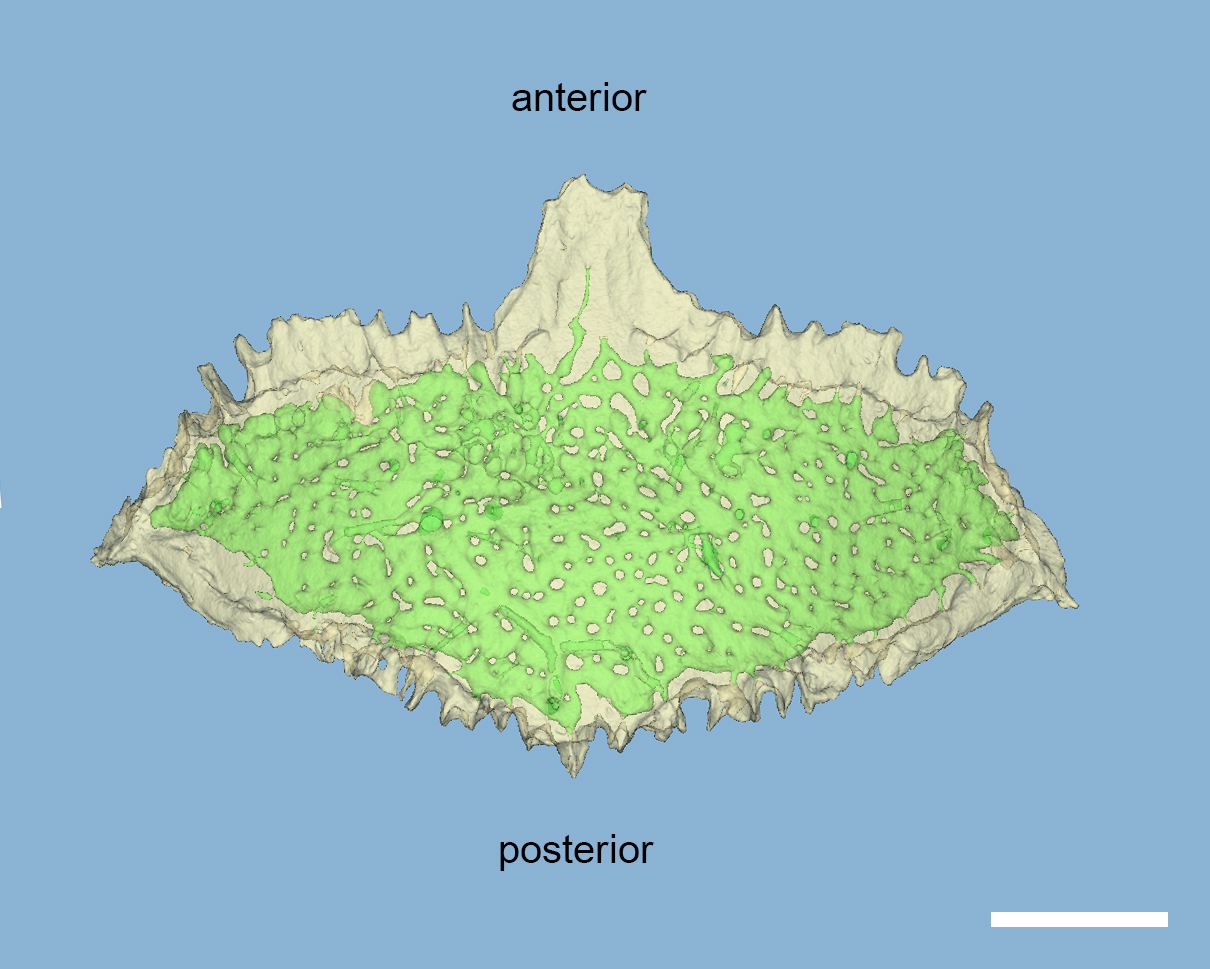

Supplement: Supplementary file 1 [file Presentation_1.zip › Supplementary Figure 1.jpeg]

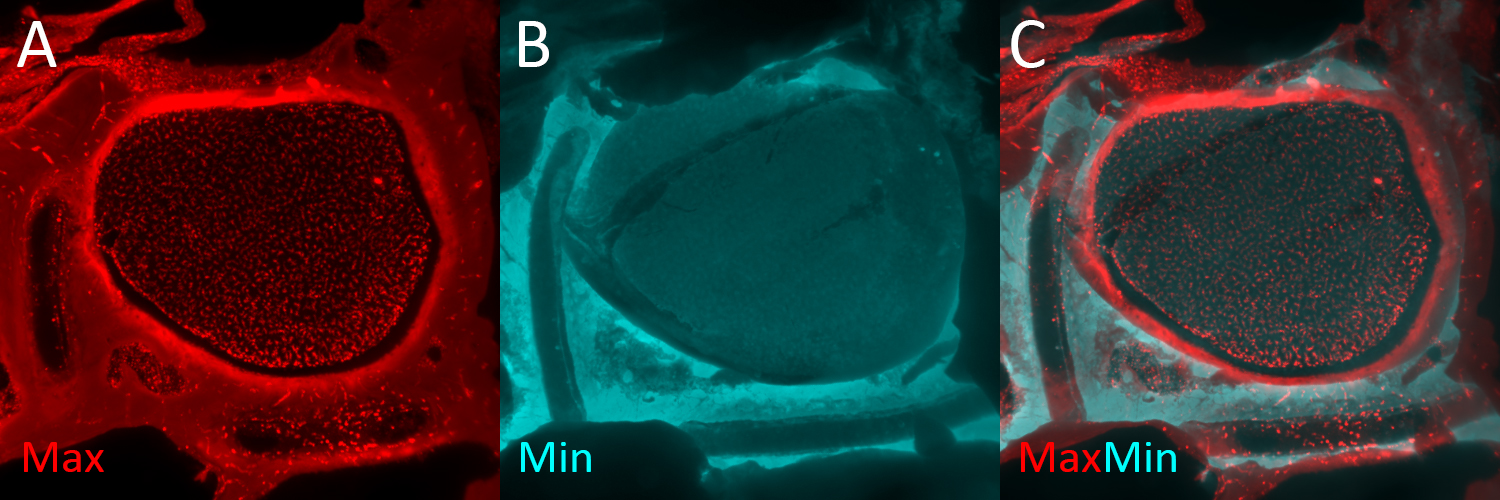

Supplement: Supplementary file 1 [file Presentation_1.zip › Supplementary Figure 2.jpeg]
